# Photoactivatable Cyclometalated Ir(III) Compound Penetrates the Blood-Brain Barrier in 3D Spheroidal and Advanced 3D Organoid Models of Inherently Resistant and Aggressive Brain Tumors

**DOI:** 10.1021/acsptsci.5c00145

**Published:** 2025-06-30

**Authors:** Vojtech Novohradsky, Alicia Marco, Marie Svitelova, Natalia Cutillas, José Ruiz, Viktor Brabec

**Affiliations:** † Czech Academy of Sciences, Institute of Biophysics, Kralovopolska 135, CZ-61200 Brno, Czech Republic; ‡ Departamento de Química Inorgánica, Universidad de Murcia and Institute for Bio-Health Research of Murcia (IMIB-Arrixaca), Murcia E-30100, Spain; § Department of Biophysics, Faculty of Science, Palacky University, Slechtitelu 27, 779 00 Olomouc, Czech Republic

**Keywords:** iridium complexes, human brain glioblastoma, 3D spheroids, blood-brain barrier penetration, ROS-mediated cytotoxicity, photodynamic therapy

## Abstract

The blood-brain barrier represents a significant challenge in delivering anticancer drugs for glioblastoma treatment. The study investigates the potential of a series of octahedral photoactivatable cyclometalated iridium complexes (**Ir1**–**Ir10**) with the general formula [Ir­(ttpy)­(C^∧^N)­Cl]­PF_6_ as photoactivated therapy candidates for the treatment of this aggressive tumor. These complexes, which include the terdentate ligand 4′-(p-tolyl)-2,2′:6′,2″-terpyridine (ttpy), and a C^∧^N ligand based on the deprotonated 2-arylbenzimidazole backbone, were tested on human glioblastoma using 2D cell cultures and 3D spheroidal models, including a fusion system comprising cerebral organoids from nonmalignant human-induced pluripotent stem cells and spheroids derived from malignant brain cells. The iridium complexes catalyze NADH photooxidation and photogenerate ^1^O_2_ and/or ^•^OH under blue light irradiation. Blood-brain barrier penetration was assessed using various *in vitro* models. The complex **Ir4**, containing deprotonated methyl 1-butyl-2-phenylbenzimidazolecarboxylate, shows promise for targeted therapy of resistant brain tumors when photoactivated with blue light. **Ir4** induces rapid and sustained ROS-mediated cytotoxicity and selectively accumulates in tumor tissue. This suggests its potential for fluorescently guided-PDT cooperative resection of glioblastoma. Notably, **Ir4** significantly reduces glioblastoma growth even under dark conditions compared to conventional Temozolomide treatment without affecting healthy brain tissue.

## Graphical abstract

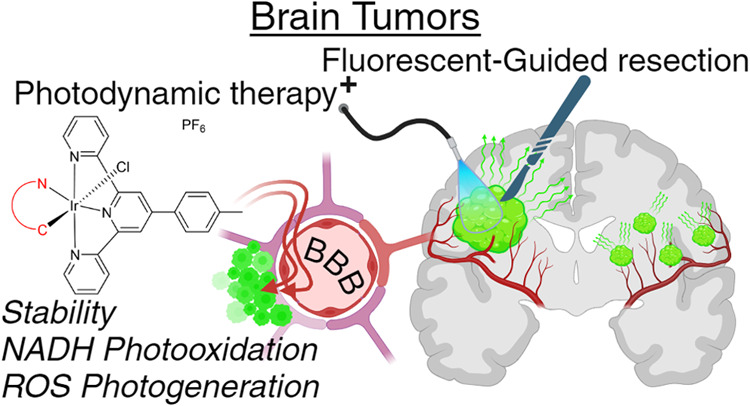

The treatment of aggressive brain tumors, such as glioblastomas, presents one of the most formidable challenges in contemporary oncology.^1,2^ Despite advances in surgery, radiotherapy, and chemotherapy, the prognosis for patients remains dismal, with median survival times rarely exceeding 15 months postdiagnosis.^3,4^ The blood-brain barrier (BBB) is central to this challenge, a highly selective physiological barrier that protects the central nervous system but significantly limits the delivery of therapeutic agents to the brain.^5^ Efforts to develop treatments capable of overcoming this barrier have included a range of strategies, from nanotechnology-based drug delivery systems to focused ultrasound and chemical modification of therapeutic agents. Nevertheless, the success of these approaches in clinical applications has been limited, necessitating the development of innovative solutions.

Among emerging therapeutic strategies, photoactivatable compounds, including metallodrugs, have garnered significant interest due to their ability to exert spatiotemporally controlled cytotoxic effects. The ability of antitumor metallopharmaceuticals to cross the BBB and effectively target glioblastoma has been demonstrated infrequently. For instance, recent studies indicate that cyclic ruthenium-peptide conjugates show promise as candidates for photoactivated therapy, exhibiting remarkable antitumor effects against glioblastoma while also possessing BBB penetration capability.^6^

Cyclometalated Ir­(III) complexes, in particular, represent a promising class of photoactivatable agents due to their unique photophysical properties, including strong absorption in the visible range, high photostability, and the ability to generate cytotoxic reactive oxygen species (ROS) upon light activation.^7–13^ Recent studies have demonstrated the potential of these compounds in targeting a variety of cancer types; however, their application in brain tumors has been relatively unexplored, particularly in advanced 3D models that better recapitulate the tumor microenvironment and the complexity of BBB penetration.

The advent of three-dimensional (3D) spheroidal and organoid models has revolutionized preclinical cancer research, offering systems that more accurately mimic the architecture, cellular heterogeneity, and physiological characteristics of *in vivo* tumors. In the context of brain cancer, such models are instrumental in assessing therapeutic efficacy and penetration through the BBB, as they can integrate critical features such as vascular mimicry and tumor-stroma interactions. These advanced models are indispensable for evaluating novel compounds that aim to address the limitations of current therapeutic strategies.

This study focuses on a novel cyclometalated Ir­(III) compound with photoactivatable properties, investigating its ability to cross the BBB and exert therapeutic effects in inherently resistant and aggressive brain tumors. Using state-of-the-art 3D spheroidal and organoid models, this work aims to elucidate the compound’s mechanistic efficacy, including its ability to generate ROS, induce apoptosis, and overcome resistance mechanisms within the tumor microenvironment. By leveraging advanced experimental models, this research provides critical insights into the translational potential of photoactivatable Ir­(III) complexes for treating malignant brain tumors, representing a significant step forward in developing innovative therapeutic strategies for one of the most challenging oncological landscapes (Scheme 1).

**Scheme 1:**
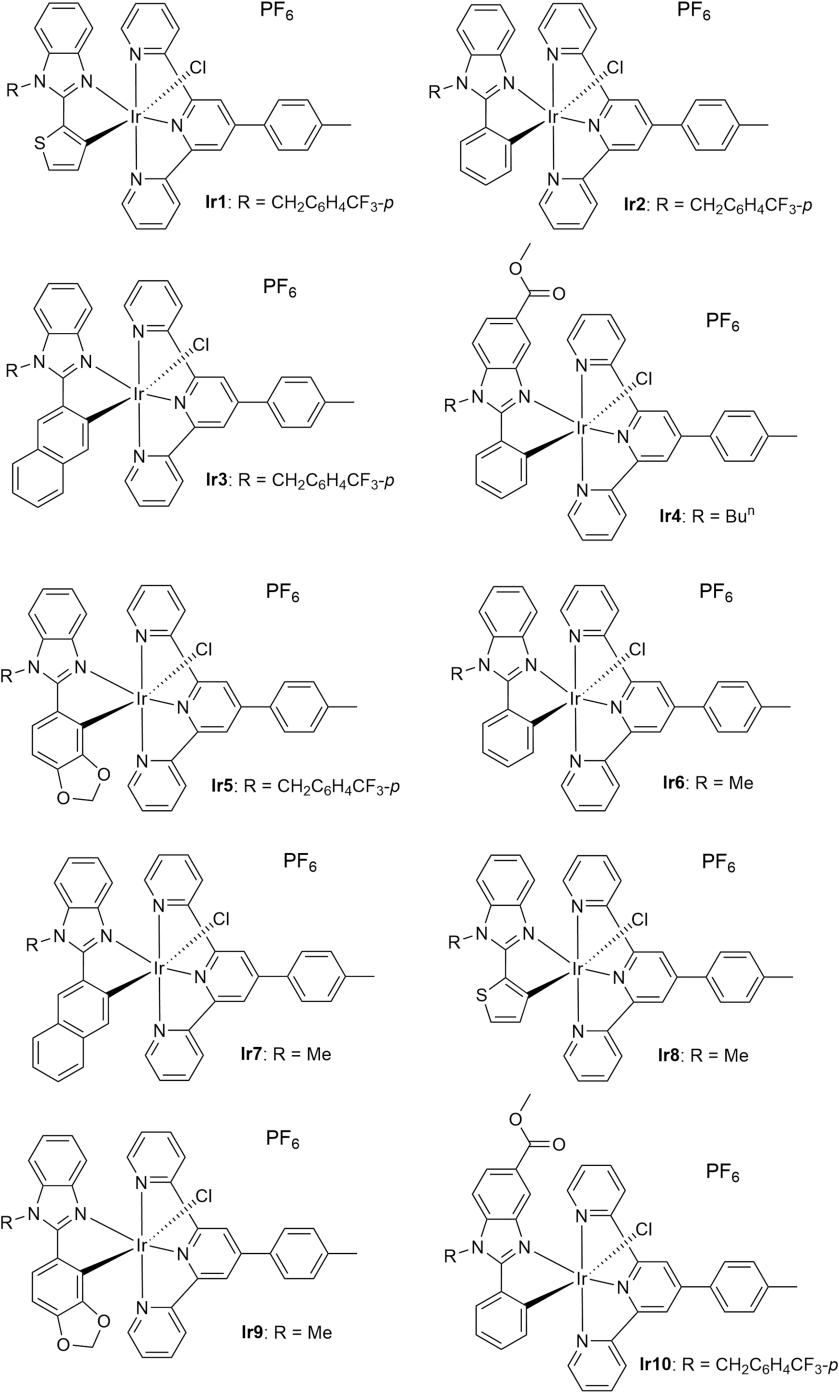
Structures of the Investigated Iridium Complexes

## Results

### Characterization of the Investigated Iridium Complexes

Phosphorescent iridium compounds, **Ir1**–**Ir9**, were previously reported.^14^ The new complex **Ir10** was synthesized in 62% yield, following a known synthetic protocol^14–16^ from the reaction of Ir­(ttpy)­Cl_3_ with the new proligand **HL10** (Schemes S1–S3). The photophysical properties of the iridium­(III) metal complexes were investigated to evaluate their potential as photosensitizers for photodynamic therapy (PDT). The absorption spectrum of the new complex **Ir10** was recorded in acetonitrile and water (Figure S18), while those of complexes **Ir1**–**Ir9** were previously reported.^14^ All complexes exhibited intense high-energy absorption bands in the 250–350 nm range due to π–π* electronic transitions on the terpyridine and cyclometalating ligands. The broad bands at 350–430 nm could be attributed to metal-to-ligand charge transfer (MLCT) and ligand-to-ligand charge transfer (LLCT) transitions. In contrast, the weak absorption shoulders at ∼450 nm are considered spin-forbidden ^3^MLCT/LLCT transitions, resulting from the spin–orbit coupling of an Ir­(III) heavy atom, which facilitates fast and efficient intersystem crossing (ISC) to convert singlet excitons to triplets. The triplet nature of these excited states could make them suitable for bioimaging and PDT. As observed, subtle structural modifications of the C^∧^N ligand only moderately affected the UV/vis absorption spectra of the corresponding Ir­(III) complexes in acetonitrile and water. Notably, upon excitation at approximately 370 nm, the complexes exhibited yellow to red emissions in both aerated acetonitrile and water (1% DMSO). The photophysical properties of complexes **Ir1**, **Ir3**, **Ir4**, **Ir5**, and **Ir10** in deaerated acetonitrile are shown in Table S1. The intense phosphorescence of **Ir3, Ir4**, and **Ir10** (Table S1 and Figures S20 and S21) could aid in tracking their intracellular localization, whereas for **Ir1**, the quantum yield was less than 10% under the same conditions. The possible aggregation-induced emission (AIE) and aggregation-caused quenching (ACQ) effects of photosensitizers were evaluated in DMSO/water mixtures with varied water volumetric fractions. Taking complex **Ir4** as an example (Figure S20), high luminescence was detected in pure DMSO. Upon increasing the fraction of water to 30%, the emission intensity increased. However, a decrease in emission was observed at 70%, along with a hypsochromic shift of the emission wavelength. A similar behavior was observed for complex **10** (Figure S21). **Ir10** was stable in DMSO, water, cell culture medium (RPMI), and in the presence of glutathione in the dark for up to 24h (Figure S19). A similar behavior was observed for complexes **Ir1**–**Ir9**.^14^

### Photochemical and Photocatalytic Properties of Iridium­(III) Complexes **Ir1**–**Ir10**

Photostability is essential for PDT agents, as photobleaching can compromise their effectiveness. The photostability of iridium complexes **Ir1**–**Ir10** was assessed in DMSO using UV/vis spectroscopy (Figure S22), revealing excellent stability under 465 nm light irradiation (4.8 mW cm^–2^) at 25 °C, indicating their potential as stable photocatalysts and photosensitizers for cellular applications. Nicotinamide adenine dinucleotide (NADH), a key electron source in the mitochondrial electron transport chain (ETC),^17^ has emerged as a target for cancer drug development,^18^ as its depletion can disrupt the hypoxic balance in cancer cells.^8,15,16,19^ The photocatalytic oxidation of NADH by complexes **Ir3–Ir5** and **Ir10** was monitored spectroscopically, showing a significant decrease in NADH absorbance at approximately 339 nm under blue light (λ = 465 nm, 4.8 mW cm^–2^)^15^ (Figure S23). Control experiments in the absence of Ir­(III) complexes (Figure S24) or light (Figure S25) corroborated the photocatalytic character of this transformation. The formation of NAD+ was confirmed by the increase in absorbance at around 265 nm, with **Ir3** demonstrating the highest photo-oxidation rate, achieving a Turnover Frequency (TOF) of about 110 h^–1^ (Table S2). Additionally, singlet oxygen (^1^O_2_) is the primary cytotoxic species in Type II PDT.^8,15^ The ability of complexes **Ir3**–**Ir5** and **Ir10** to generate singlet oxygen was evaluated by measuring the decrease in 1,3-diphenylbenzofuran (DPBF) absorbance at 411 nm under blue light irradiation (Figure S26). The iridium complexes exhibited high singlet oxygen quantum yields, with **Ir3** reaching a yield of up to 95%, indicating their suitability as photosensitizers for PDT. Furthermore, the generation of hydroxyl radicals (^•^OH) was assessed using hydroxyphenyl fluorescein (HPF) as a probe, which exhibited increased fluorescence intensity upon exposure to blue light.^20^ This confirmed the production of hydroxyl radicals, particularly with complexes **Ir3** and **Ir10**, after 8 min of irradiation (Figure S29). In summary, the iridium complexes exhibit promising photostability, effective NADH oxidation, and significant generation of singlet oxygen and hydroxyl radicals, supporting their potential application in photodynamic therapy for cancer treatment.

### Initial Biological Screening Studies for Dosing Optimization in Advanced Spheroid and Organoid Models

#### Antiproliferative Effects in 2D Monolayer and 3D Spheroid Models of Brain Tumor Cell Cultures

Evaluating antiproliferative activity is essential for characterizing anticancer compounds and optimizing drug candidates. This study utilized the brain cancer cell line U87MG (glioblastoma model).^21^ While 2D models are easier, cheaper, and faster to use, their limitations often result in poor translation of findings to clinical outcomes. On the other hand, 3D spheroid models offer a more accurate and predictive platform for studying antiproliferative effects and drug efficacy, albeit at a higher complexity and time consumption.^22^

We assessed the antiproliferative activity of the iridium complexes in both 2D monolayers and 3D spheroids under dark and irradiated conditions (Table S4).

Results describing the antiproliferative activity of the tested compounds in 2D monolayers are often used to estimate the therapeutic window. Therefore, we first conducted preliminary experiments to identify clinically relevant parameters. Specifically, we assessed three irradiation durations (10, 20, and 30 min) using blue light (420 nm, 58 W m^2–^). Our results demonstrated a strong dependence of phototoxic activity on irradiation time, with a 10 min reduction typically leading to an approximate 50% decrease in phototoxicity. At 10 min, negligible photoactivation was observed under the selected conditions. Conversely, significantly longer durations were deemed impractical for clinical application.

Based on these findings, a 30 min exposure was selected for the detailed experiments reported in the manuscript, as it provided a suitable balance between efficacy (i.e., phototoxic index and cytotoxicity) and practical feasibility. While comprehensive data from these optimization studies were collected, we focused the manuscript on results obtained under the selected conditions to maintain clarity and conciseness.

IC_50_ values (concentration of the compound reducing cell growth by 50%) for U87MG cells in the dark were in the range of 4.3–34 μM; they dropped significantly after irradiation (IC_50_ values were in the range of 0.4–2.2 μM), indicating considerable sensitization. 3D spheroids showed reduced antiproliferative activity even after irradiation, with IC_50_ values ranging from 2.1 to 10.5 μM, likely due to poor penetration of the iridium complexes into deep-seated cells in the spheroids (**Ir1**–**Ir3**, **Ir5**–**Ir10**). Tumor mass indexes (TMIs) indicated varying reductions in activity between 2D and 3D models, with **Ir4** showing the lowest TMI of 4.

#### Analysis of Morphologic Parameters of U87MG Spheroids after Treatment and Irradiation with the Investigated Compounds

We further investigated the effects of treatment and irradiation on tumor spheroids by analyzing the morphology and diameter of spheroids from the U87MG cell line. Morphogenic parameters were assessed using phase contrast microscopy at 24-h intervals during the preformation phase and post-treatment, culminating at 144 h. Initial mean diameters of the spheroids were 280 ± 11 μm, increasing to 550 μm after 70 h of drug-free incubation. U87MG spheroids proliferated rapidly. Rapidly growing tumor cells are generally more sensitive to treatment, whereas quiescent tumor cells are moderately sensitive to metal-based compounds.^23^

The spheroid models underwent treatment with varying concentrations of compounds, focusing on equitoxic concentrations during blue light exposure. The analysis included both irradiated and nonirradiated samples to assess nonspecific toxicity under dark conditions. U87MG spheroids showed a lighter outer layer and an initial optically dense core, indicating early necrotic core formation. Significant morphological changes were observed after 90 min treatment with compounds and subsequent 30 min blue light irradiation and 70 h of drug-free incubation. U87MG spheroids tended to lose their dense structure, leading to disaggregation, initiated from the luminal border. The most pronounced effects were observed after treatment with Ir4, where spheroid diameters decreased below their initial size, suggesting cytostatic effects and systemic toxicity that inhibited further growth of U87MG spheroids after treatment.

The iridium complexes (except **Ir4**) had a moderate impact on spheroid growth, which was more significant than that of clinically used Temozolomide for brain tumors (Figure S30). Given the known resistance of brain tumors to chemotherapeutics,^24,25^ **Ir4**, and in some cases, **Ir3** and **Ir5**, were selected for further mechanistic studies on phototherapeutic intervention based on a comprehensive evaluation of the antiproliferative activity and phototoxic properties of the investigated Ir complexes across both 2D monolayer cultures and 3D spheroid models (Table S4, Figure 1, panel I, and Figure S30). The selection prioritized compounds exhibiting the highest photoactivation potential, a key prerequisite for the mechanistic studies presented in this work. Despite model-dependent variability, **Ir3**, **Ir4**, and **Ir5** consistently showed the most robust and significant photoactivation across both platforms. U87MG glioblastoma models were also chosen for further investigations due to glioblastoma’s incurable nature.^26^

**Figure 1:**
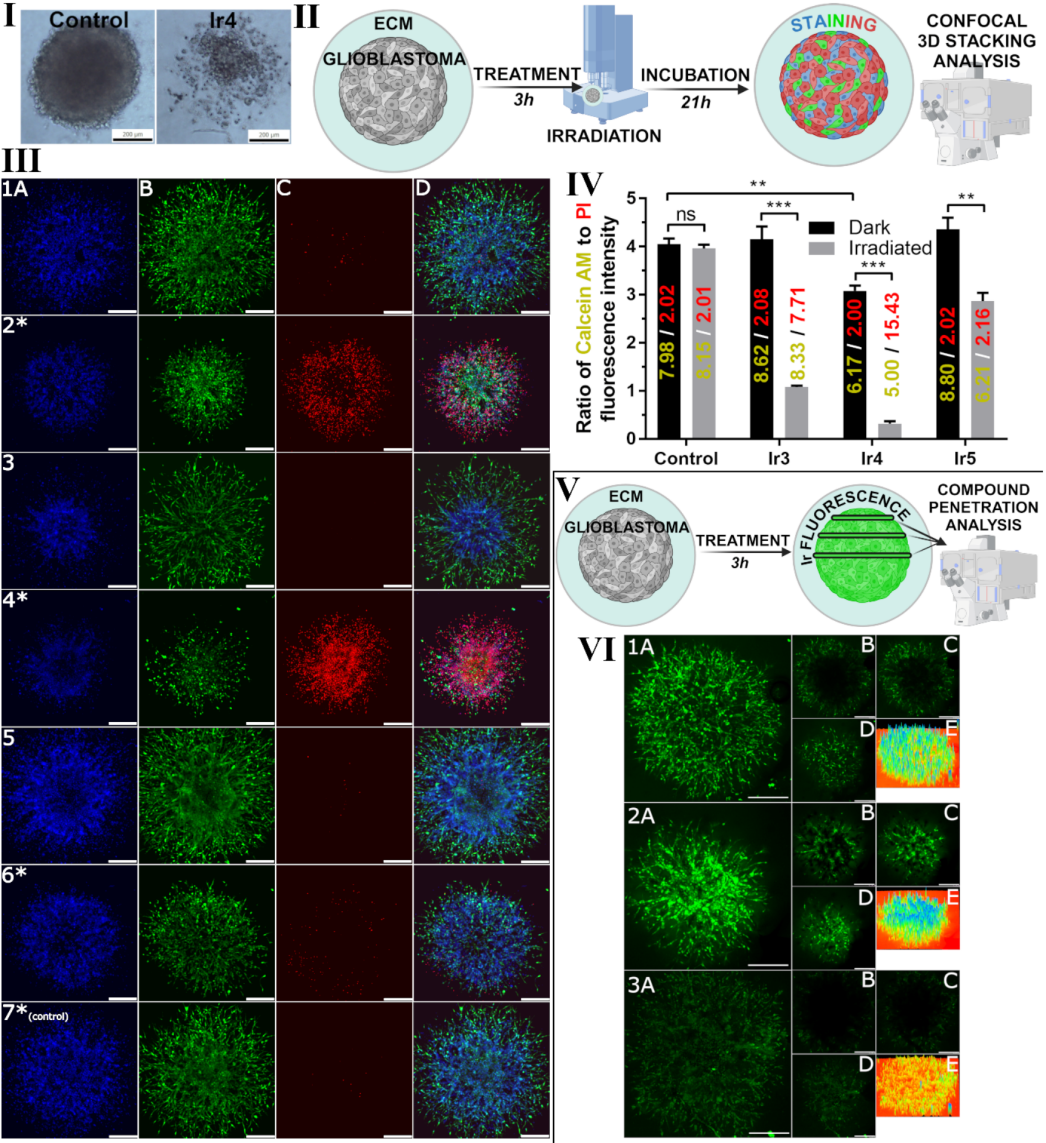
Evaluation of spheroid morphology, drug penetration, and cellular response of glioblastoma spheroids embedded in extracellular matrix to iridium-based compounds. **I**, Analysis of the morphology of the brain glioblastoma U87MG spheroids treated with **Ir4** or untreated control, both irradiated with blue light. Scale bar 200 μm. **II**, Schematic representation of the experimental methodology for investigating the drug response of ECM-glioblastoma spheroids (ECM = extracellular matrix) to selected iridium-based compounds. **III**, Analysis of spheroids after irradiation with 405 nm blue light by confocal microscopy. Spheroids were treated for 3 h with equitoxic concentrations of **Ir3**, **Ir4**, and **Ir5**, corresponding to the IC_50_s determined for irradiation samples, and then irradiated with 405 nm laser light (1 mW, 60 s). After 21 h post-treatment and irradiation, the samples were stained with (A) Hoechst 33258 dye, (B) calcein AM, and (C) propidium iodide (PPI). Merged channels are in panel D. Samples: panels 1, 2*—**Ir3**; panels 3, 4*—**Ir4**; panels 5, 6*—**Ir5**. Irradiated control is shown on panel 7*. Samples irradiated with 405 nm laser light are marked with stars. Images are the maximal projections of the 3D *z*-stacks, and the representatives of three independent experiments are shown. Scale bars represent 200 μm. **IV**, Quantification of Calcein AM to PPI fluorescence intensity ratio analyzed from the confocal microscopy microphotographs. The initial values of the mean fluorescence intensities for the ratio calculation are shown in the bars, with green values representing calcein AM intensities and red values representing PPI intensities. Data were subjected to statistical analysis using the Student’s *t* test with the following significance: **p ≤ 0.01; ***p ≤ 0.001; ns = nonsignificant. Data are the means from three independent experiments ± SDs. **V**, Schematic representation of the experimental methodology for investigating drug penetration into extracellular matrix-embedded glioblastoma spheroids. **VI**, Distribution of the investigated Ir complexes in U87MG glioblastoma spheroids embedded in Matrigel matrix analyzed by confocal microscopy. Samples were treated for 3 h with 5 μM of **Ir3** (panel 1), **Ir4** (panel 2), and **Ir5** (panel 3). *Z*-stack fluorescence intensity projections from tested compounds are on the corresponding panels A, whereas panels B, C, and D are the separate *z*-stacks from the mid, upper-mid, and upper part of the spheroid. Panel E shows the 3D surface plot of the *z*-stack maximal fluorescence intensity projection. Scale bars represent 300 μm.

### Effects of Iridium Complexes and Blue Light on U87MG Spheroid Metabolism and Morphology

Our study investigated the impact of iridium complexes (**Ir3**–**Ir5**) combined with blue light irradiation on three-dimensional spheroids derived from U87MG glioblastoma cells, which were embedded in a Matrigel matrix. Using confocal microscopy and calcein AM-PPI staining, we assessed cell viability within the spheroids (Figure 1, panel III). Hoechst staining defined spheroid contours, enabling the application of a Region of Interest (ROI) mask for fluorescence intensity analysis and Calcein AM to PPI ratio quantification (Figure 1, panel IV). This approach enables the evaluation of cell viability, drug uptake, and diffusion in 3D spheroid models under controlled irradiation conditions.^27^ Methodological details for calcein AM/PPI staining and drug penetration studies are summarized in Figure 1, panels II and V.

Spheroids were treated with iridium complexes at IC_50_ concentrations (Table S4) for 3 h, followed by 405 nm laser irradiation for 60 s. After 24 h in a drug-free medium, spheroids were stained for analysis. Significant differences in live and dead cell counts were observed between irradiated and nonirradiated samples for **Ir3** and **Ir4**, with morphological disruptions noted in treated spheroids, including reduced diameters and metabolically active cells concentrated in the core (Figure 1, panel III, inserts 2* and 4*). **Ir5**-treated samples showed minimal changes in metabolic activity or PPI staining (Panel III, Inserts 5* and 6*), likely due to reduced **Ir5** accumulation in spheroids (Figure 1, panel VI).

**Ir3** and **Ir4** demonstrated significant penetration into U87MG spheroids, with **Ir4** fully penetrating the core. Quantitative fluorescence analysis showed insignificant changes in calcein AM/PPI ratios for **Ir3** and **Ir5** under dark conditions, while a significant decrease was observed for **Ir4**-treated spheroids, indicating reduced metabolic activity. The most pronounced effects were observed with **Ir3**, **Ir4**, or **Ir5** combined with blue light irradiation, resulting in a substantial decrease in calcein AM/PPI ratios due to a reduction of metabolically active cells and an increase in dead cells. **Ir4** exhibited the most potent effects, highlighting its superior photoactivity in targeting glioblastoma cells. These findings suggest the potential efficacy of iridium complexes in targeted glioblastoma treatments and warrant further investigation into their mechanisms and therapeutic applications.

### Distribution of the Investigated Iridium Compounds in Matrigel-Embedded U87MG Spheroids by Confocal Microscopy

Studying the distribution of anticancer agents in advanced *in vitro* models, such as spheroids, is essential for understanding tumor cell behavior and drug responses.^28^ Spheroids serve as physiologically relevant alternatives to conventional 2D cell cultures, closely replicating the *in vivo* tumor microenvironment. This multicellular structure provides valuable insights into drug penetration, metabolism, and distribution, which are critical for predicting therapeutic efficacy and resistance.^28,29^ Additionally, embedding spheroids in extracellular matrices, such as Matrigel, facilitates the evaluation of drug responses in three-dimensional settings, better reflecting the influence of the tumor microenvironment on drug activity.^30^ The interaction between glioblastoma cells and the ECM further enhances spheroid invasiveness, underscoring the relevance of this model.

To investigate the spatial distribution of the tested compounds, we employed a more complex system involving Matrigel-embedded spheroids, rather than simple, nonembedded spheroids cultured under ultralow attachment conditions (Figure 1, panels V and VI). The embedded spheroids exhibited distinct morphological differences compared to unembedded models (see Figure S30 for comparison).

In our experiments, spheroids treated with various iridium-based compounds (**Ir3**, **Ir4**, and **Ir5**) exhibited distinct fluorescence patterns, indicating differential penetration and distribution within the spheroid mass. Notably, **Ir4** displayed the most uniform distribution, suggesting a strong correlation between penetration and anticancer activity. A faint extracellular fluorescence was also observed in the Matrigel matrix, particularly with **Ir4** treatment. **Ir3** and **Ir5** were primarily localized in the outer regions of the spheroids, within a 300-μm envelope, whereas **Ir4** achieved a more homogeneous distribution with minimal fluorescence-free areas in the core. These results, combined with the favorable photoactivable properties of **Ir4**, highlight its effectiveness, as reflected in its antiproliferative activity (Table S4).

### Detection of Reactive Oxygen Species (ROS) in U87MG Spheroids via Confocal Microscopy

ROS play a pivotal role in PDT, a treatment modality in which a photosensitizer is activated by light, leading to the generation of ROS and subsequent cell death.^31^ PDT offers several advantages, including minimal systemic toxicity and the ability to overcome multidrug resistance.^31^ Furthermore, combining ROS-responsive chemotherapy with PDT and laser-based imaging has shown promise in enhancing therapeutic outcomes.^32^

Preliminary screening of compounds **Ir3**, **Ir4**, and **Ir5** identified **Ir4** as the most promising candidate for glioblastoma treatment. Its selection for subsequent in-depth studies was based on its superior performance in spheroid penetration, inhibition of metabolic activity, induction of cell death, and favorable apparent permeability (*P*_app_) across the BBB (see sections Results, Effects of Iridium Complexes and Blue Light on U87MG Spheroid Metabolism and Morphology, Distribution of the Investigated Iridium Compounds in Matrigel-Embedded U87MG Spheroids by Confocal Microscopy, and Blood-Brain Barrier Penetration Assessed Using a 2D In Vitro BBB Model with hCMEC/D3 Cells).

We evaluated ROS induction in glioblastoma U87MG spheroids treated with **Ir4** at its IC_50_ concentration for 3 h, followed by blue laser light irradiation (405 nm, 60 s, 1 mW). ROS levels were quantified using the CellRox probe, with PPI employed to assess immediate cell death. *Z*-stack imaging was performed before irradiation and at 1, 15, and 30 min postirradiation (Figure 2, panel I). Mean fluorescence intensity in the *z*-stack projections quantified ROS generation (Figure 2, panel II).

**Figure 2:**
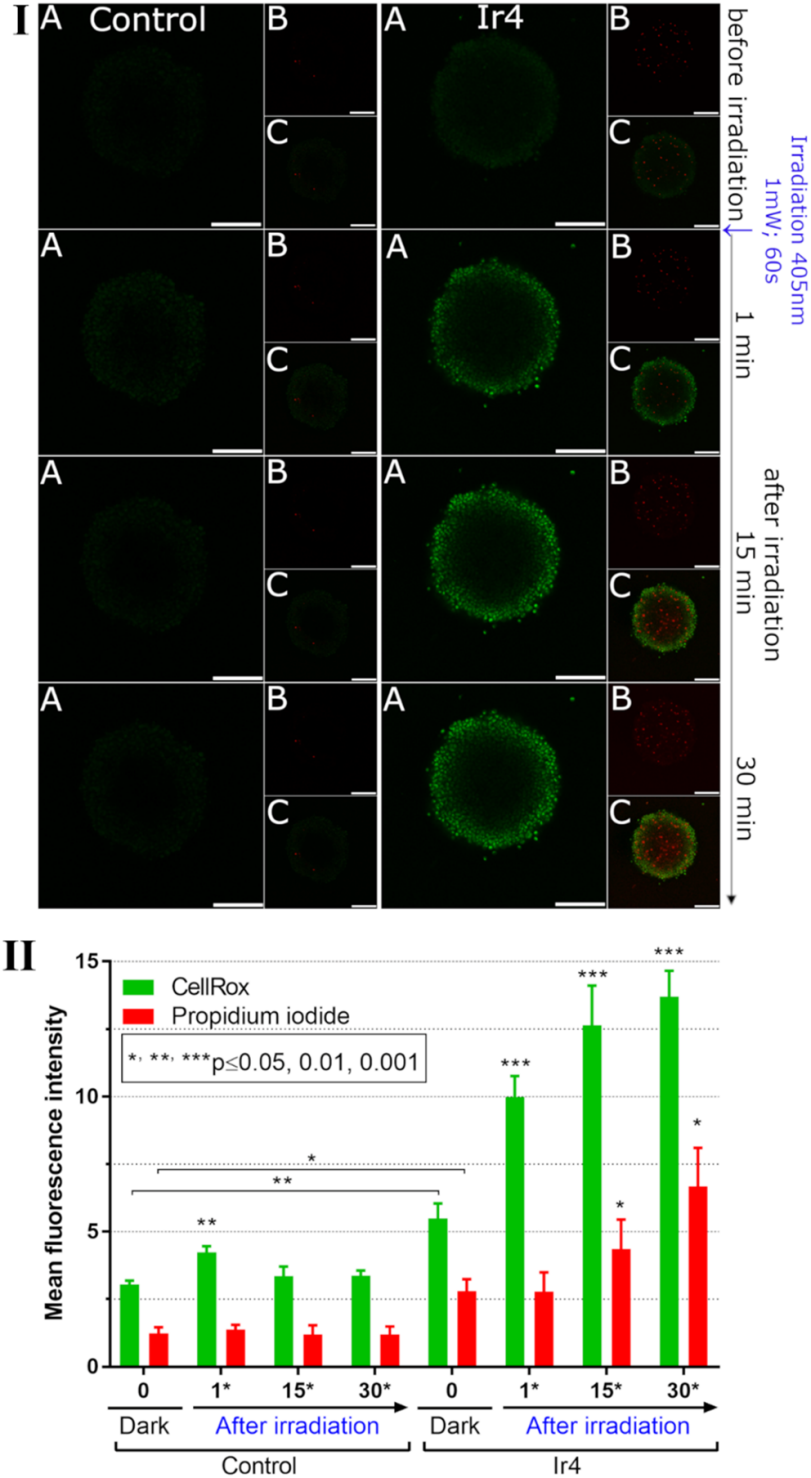
ROS generated by blue light irradiation of glioblastoma spheroids treated with **Ir4**. **I**, Detection of ROS in U87MG glioblastoma spheroids by confocal microscopy. Spheroids were treated with **Ir4** (IC_50, U87MG 3D, 72 h)_ for 3 h or kept under drug-free conditions (control). Samples were then stained with CellRox reagent and PPI and irradiated for 60 s with blue laser light (405 nm, 1 mW). Samples were scanned immediately after the irradiation (1 min) and then 15 and 30 min afterward. Panel A: fluorescence coming from CellRox, panel B: PPI fluorescence, panel C: the overlay of the CellRox and PPI fluorescence. Images represent a maximal projection of 10 *z*-stacks; scale bar 300 μm. **II**, Quantification of the mean fluorescence intensity of the CellRox and PPI in U87MG spheroids irradiated with blue laser light. Samples were treated for 3 h with **Ir4** at the concentration corresponding to IC_50,U87MG 3D,72h_.

Before laser irradiation, **Ir4**-treated spheroids exhibited ROS levels 1.8 times higher than untreated controls, suggesting that ROS generated under dark conditions contributed to reduced metabolic activity, as evidenced by calcein AM staining (Figure 1, panels III and IV). Following laser irradiation, ROS levels in **Ir4**-treated samples increased steadily, reaching 1.8-, 2.3-, and 2.5-fold compared to preirradiation levels at 1, 15, and 30 min, respectively. In contrast, untreated controls exhibited only a transient 1.4-fold increase in ROS immediately after irradiation, with negligible changes thereafter.

Notably, a slight but significant increase in PPI fluorescence intensity was detected after 15 and 30 min postirradiation, indicating the onset of rapid cell death as a direct result of ROS generation. These findings highlight **Ir4**’s potential to induce sustained, ROS-mediated cytotoxicity, underscoring its promise for further development in cancer therapy.

### Blood-Brain Barrier Penetration Assessed Using a 2D *In Vitro* BBB Model with hCMEC/D3 Cells

Understanding blood-brain barrier (BBB) permeability is essential for advancing brain tumor imaging and therapeutics. Among various *in vitro* BBB models,^33,34^ those based on immortalized human cerebrovascular hCMEC/D3 cells offer potential for standardized application.^35–38^ In this study, a BBB model was established by seeding 5 × 10^4^ hCMEC/D3 cells onto 24-well tissue culture inserts (3 μm pore size, 0.3 cm^2^ surface area) precoated with collagen I. Cells were cultured for 7 days to facilitate complete differentiation and the formation of a barrier. Monolayer integrity was assessed via trans-endothelial electrical resistance (TEER),^39^ measured continuously for 48 h post-treatment with test compounds (10 μM) under dark conditions. TEER values ranged from 53 to 80 Ω·cm^2^, consistent with previously reported values exceeding 50 Ω·cm^2^ for fully developed hCMEC/D3-based *in vitro* BBB models.^35,36,38^

Following model characterization, *P*_app_ values were determined to evaluate differences in BBB penetration among the test compounds (Table 1). The Transwell migration assay^40^ was used to establish *P*_app_ values and compare them with those of the clinically utilized Temozolomide. Iridium compound concentrations in the apical and basolateral compartments were quantified via inductively coupled plasma mass spectrometry (ICP-MS). *P*_app_ values were calculated using the formula: *P*_app_ = (d*C*/d*t*)·(*V*_r_/*AC*_0_), where d*C*/d*t* represents the cumulative concentration of the iridium compound in the basolateral compartment over time, *V*_r_ is the compartment volume (1.5 mL), A is the surface insert area (0.336 cm^2^), and *C*_0_ is the initial apical concentration.^40^

**Table 1:** Apparent Permeability Coefficients Measured on the Model of hCMEC/D3 Cells

compound^a^	*P*_app_ × 10^–4^ (cm·s^–1^)^b^
**Ir3**	0.21 *±* 0.05
**Ir4**	3.4 *±* 0.2
**Ir5**	0.19 *±* 0.04
Temozolomide^c^	0.36

a The initial concentration of tested iridium compounds in the apical part was 10 μM.

b The data are the means ± SDs from three independent experiments with duplicates for each experimental run.

c Data taken from the ref 41.

Results revealed significant differences in BBB permeability among the tested iridium compounds. *P*_app_ values for **Ir3** and **Ir5** were at least an order of magnitude lower than those for **Ir4**, indicating substantially reduced BBB penetration. Given the therapeutic interest in metal-based anticancer compounds capable of crossing the BBB, these findings suggest that **Ir4** exhibits superior permeability, surpassing even that of Temozolomide, thus highlighting its potential for brain cancer treatment.

### Blood-Brain Barrier (BBB) Penetration Investigated Using the Transwell Binary Coculture BBB Model

The cellular uptake of anticancer compounds after penetrating the BBB is crucial for their efficacy in treating brain tumors. Understanding the extent of cellular uptake provides insights into the distribution and localization of these compounds within brain tumor cells, making such studies essential for assessing their potential efficacy.^42^ We utilized the static Transwell binary coculture BBB model,^36^ comprising an epithelial hCMEC/D3 cell line (representing the BBB) and a glioblastoma U87MG cell line. In this model, hCMEC/D3 cells were seeded on the apical side of the Transwell, while U87MG cells were seeded on the basolateral side. The compound **Ir4**, at a concentration of 10 μM, was applied to the apical side to simulate penetration from the blood to the brain interstitial fluid. After a 24-h incubation, cells were harvested, counted, and the iridium uptake by both cell lines was analyzed using ICP-MS. The iridium uptake from Ir4 by hCMEC/D3 and U87MG cells in the binary coculture BBB model was 47 ± 7 ng Ir/10^6^ cells and 108 ± 14 ng Ir/10^6^ cells, respectively (mean ± SD from three independent experiments). These data indicate a more than 2-fold higher accumulation of **Ir4** in U87MG cells compared to hCMEC/D3 cells. Despite significant uptake by BBB-hCMEC/D3 cells, the predominant uptake by U87MG cells highlights **Ir4**’s potential efficacy in penetrating the BBB and targeting brain tumors.

### Effects of **Ir4** in the Fusion System Composed of Cerebral Organoids with Implanted Glioblastoma

Cerebral organoids, 3D stem cell-derived models mimicking the human brain, have become valuable tools for studying anticancer compounds. These organoids provide a unique platform for investigating the effects of various compounds, therapies, and factors on human-derived tissue, closely mimicking in vivo conditions while facilitating convenient *in vitro* experimentation without the need for ethical constraints.^43,44^ Studies have demonstrated the potential of cerebral organoids in modeling brain tumors, such as glioblastoma, and evaluating anticancer therapies.^45^ Additionally, cerebral organoids facilitate the prediction of therapeutic responses and the screening of potential anticancer agents, providing insights into the development and testing of therapeutic interventions.

The notable activity of **Ir4** against human glioblastoma U87MG spheroids (Table S4) does not guarantee clinical success, as *in vivo* conditions may alter drug activity. Therefore, we developed a fusion system in which nonmalignant tissue is represented by cerebral organoids derived from human induced pluripotent stem cells (hiPSCs), and U87MG implants represent malignant tissue. This system, embedded in a Matrigel capsule, allows for convenient sample manipulation, analysis, and processing. An example of such an organoid-spheroid implant (Figure S31) shows visible invasion of U87MG glioblastoma into the nonmalignant cerebral organoid. This fusion system enables the monitoring of compound distribution, treatment effects on healthy brain tissue, and tumor spread in conditions that resemble *in vivo* human scenarios.

We aimed to observe the distribution of **Ir4** within the fusion system of cerebral organoids and spheroids formed from malignant U87MG brain cells. **Ir4** predominantly accumulated in malignant tissues, with some fluorescence signal increase observed in the periphery of the nonmalignant cerebral organoid (Figure 3, panel I). Ongoing research focused on the selective accumulation of **Ir4** in tumor tissue, with findings to be published separately. These results align with the cellular accumulation patterns observed in the hCMEC/D3 and U87MG coculture model, demonstrating predominant accumulation in U87MG cells. The selective accumulation of **Ir4** in tumor tissue suggests its potential application as an effective agent for PDT and as a candidate for fluorescently guided PDT cooperative resection of glioblastoma, due to its bright fluorescence and tumor-accumulating properties.^46^ Fluorescently guided resection has shown potential in improving the extent of resection by visualizing brain tumor tissue during surgery, including in areas such as the subependymal zone.^47^

**Figure 3:**
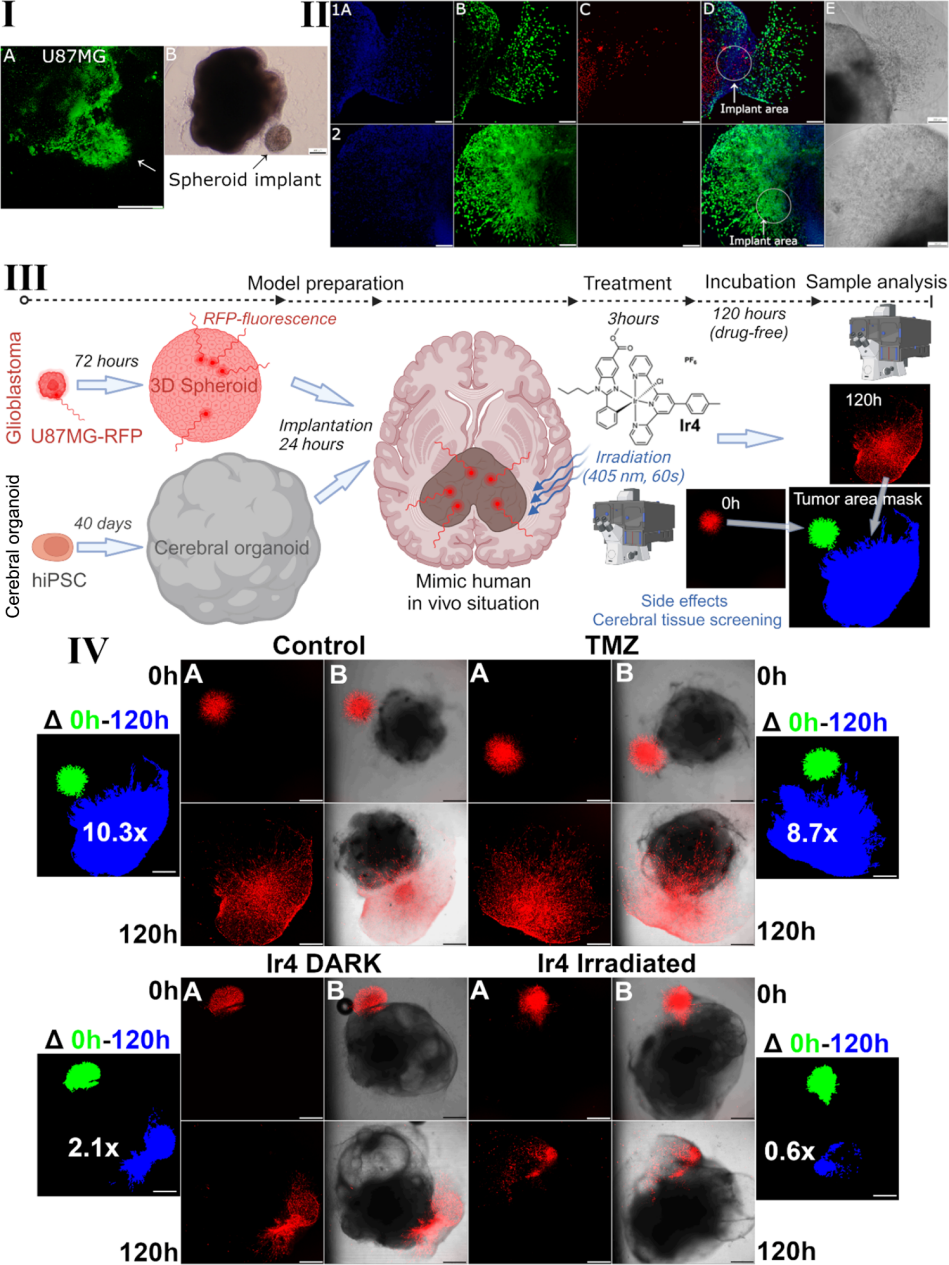
Mimicking a human *in vivo* situation. Establishment of organoid-spheroid fusion model, analysis of cancer tissue selective accumulation of **Ir4**, and cancer tissue volume analysis. **I**, Representatives of the organoid-spheroid implant model for distribution studies. Implanted spheroids were derived from U87MG glioblastoma cells, representing a model system for studying malignancy. The implant model was exposed for 3 h to 5 μM of compound **Ir4**. The fluorescence signal emitted by the tested compound **Ir4** was visualized using a confocal microscope in Panel A, allowing for precise imaging of the distributed compound. Panel B displayed a phase contrast image obtained through an optical microscope, providing additional insights into the structural characteristics of the model system. Cerebral organoids represent healthy tissue, and the spheroid implants mimic malignant behavior. Scale bars represent 200 μm. **II**, Organoid-spheroid implants after 120 h post-treatment with **Ir4** and irradiation. Generated U87MG-cerebral organoid implants were treated with **Ir4** (panels 1) or left untreated (panels 2) for 3 h following selective irradiation with blue laser light (405 nm, 60 s, 1 mW). Samples were stained with Hoechst 33258 (panel A), calcein AM (panel B), PPI (panel C), overlay of fluorescence channels (panel D), and bright-field (panel E). Fluorescence images were captured using a confocal microscope, while bright-field images were obtained using an inverted microscope equipped with phase contrast mode. The area of initial implantation of U87MG spheroid was demarcated with white circles, and the scale bar represents 200 μm. **III**, The schematic presentation outlines the methodology for tumor intergrowth and migration studies. Cerebral organoids were derived from hiPSCs and cultured for 40 days to achieve full maturity. U87MG-RFP cells (RFP = red fluorescent protein) were then cultured in 3D conditions for 72 h to form RFP-tagged spheroids. Co-implantation of healthy cerebral organoids and malignant U87MG-RFP spheroids was conducted after a 24-h incubation. Subsequently, samples were either treated with compounds or left untreated for 3 h before analysis using confocal microscopy to assess the initial state. Samples designated for irradiation underwent selective exposure to blue laser light. A drug-free period of 120 h allowed the implanted tumor spheroids to develop fully, enabling the long-term effects of treatment to be monitored. Finally, analysis by confocal microscopy revealed the expansion of glioblastoma tumors through the area occupied by RFP-tagged U87MG cells. Concurrently, healthy tissue, represented by cerebral organoids, was monitored for growth and potential side effects of treatment using confocal or bright-field microscopy. **IV**, Intergrowth and migration of U87MG-RFP cells to a cerebral organoid. U87MG-RFP spheroids were introduced into fully matured cerebral organoids 24 h before treatment with Temozolomide (TMZ; 100 μM) or compound **Ir4** (IC_50_, U87MG 3D, 72h). Following a 3-h exposure to the test compounds, the samples were irradiated with blue laser light (405 nm, 60 s, 1 mW), except for Ir4-DARK and TMZ samples, which were not exposed to blue light. The time point for an initial state was marked as time 0 h, and the experiment was terminated after 120 h post-treatment, showcasing the localization of U87MG-RFP cells at these time points. Panel A illustrates the fluorescence channel for U87MG-RFP localization, while panel B presents an overlay of the bright field and fluorescence channel. Changes in the U87MG expansion area are highlighted within the Δ*p*Anel. Fluorescence masks were pseudocolored and, for illustrative purposes, included in a single figure. The initial state of U87MG-RFP at the treatment onset (time 0) is pseudocolored in green, and the 120 h experimental end point is pseudocolored in blue. The expansion area relative to the initial state is also demarked in the panel Δ. Scale bars represent 500 μm.

### Impact of **Ir4** Treatment Combined with Blue Laser Irradiation on a Cerebral Organoid-Glioblastoma Implant Model

To evaluate the effects of **Ir4** treatment combined with blue laser irradiation on cerebral tumors, a fusion system was stained with Hoechst 33258, calcein AM, and PPI. Implant samples were treated with **Ir4** (IC_50,U87MG 3D,72h_) for 3 h, followed by blue laser irradiation (405 nm, 60 s, 1 mW), and incubated in drug-free medium for an additional 120 h to assess long-term effects, including glioblastoma invasion and proliferation. The dosing used for the glioblastoma organoid implant model was based on the IC_50_ values obtained for U87MG 3D spheroids (Table S4). Specifically, the administered dose corresponds to approximately 2 × IC_50_ against U87MG spheroids, which we believe represents a rational and clinically relevant concentration for evaluating drug efficacy in this more complex 3D model. The irradiation targeted only the U87MG implants and their contact periphery to minimize damage to nonmalignant tissue. Confocal microscopy was used to visualize the samples postincubation (Figure 3, panel II).

After 120 h postirradiation, apparent differences were observed between treated and untreated samples, particularly in calcein AM fluorescence, which reflects metabolically active cells. Untreated samples (Figure 3, panel II, inserts 2) exhibited extensive glioblastoma spread and higher metabolic activity compared to **Ir4**-treated samples (Figure 3, panel II, inserts 1), where only a few metabolically active malignant cells were present at the implantation site. This suggests that tumor spread in treated samples originated from residual cells that survived the irradiation, a phenomenon commonly encountered in clinical settings. However, tumor progression in treated samples was significantly reduced compared to untreated controls. These findings highlight the need for optimized treatment regimens, such as repeated applications or combination therapies (e.g., γ irradiation), to eliminate residual malignant cells further.

### Suppression of Glioblastoma Intergrowth into Cerebral Organoids by **Ir4** Treatment Combined with Blue Laser Irradiation

Investigating glioblastoma intergrowth into healthy tissue, modeled by cerebral organoids, is a critical area of research in neurobiology and oncology. Glioblastoma’s invasive nature and resistance to standard therapies pose significant challenges. By studying glioblastoma behavior within the complex microenvironment of cerebral organoids, researchers aim to uncover mechanisms of tumor progression and its interaction with healthy brain tissue. Advances in organoid technology have provided robust preclinical models for glioblastoma research, surpassing traditional models like patient-derived xenografts in immunodeficient mice in replicating the tumor microenvironment.^48,49^

In this study, U87MG-RFP glioblastoma spheroids were preformed over 72 h, implanted into cerebral organoid tissue, and coembedded in Matrigel. Following a 24-h stabilization period, the spheroid-organoid system was treated with Temozolomide (TMZ, 100 μM) or **Ir4** (2 μM; IC_50,U87MG 3D,irradiated,72h_) for 3 h. Samples designated for irradiation were exposed to blue laser light (405 nm, 60 s, 1 mW) and subsequently cultured in drug-free medium for 120 h. *Z*-stacking confocal imaging and ImageJ analysis quantified the area of glioblastoma spreading (Figure 3, panels III and IV).

Control samples (untreated, irradiated) exhibited a 10.3-fold increase in glioblastoma spreading area 120 h postirradiation. TMZ-treated samples showed an 8.7-fold increase, consistent with glioblastoma’s known resistance to TMZ in clinical settings. In contrast, **Ir4**-treated samples demonstrated significantly reduced glioblastoma spreading, with a 2.1-fold increase over the same period. Notably, **Ir4**-treated samples under nonirradiated conditions displayed a spreading area four times smaller than that observed with TMZ, despite the **Ir4** concentration being 50 times lower. When combined with selective blue laser irradiation, **Ir4** effectively suppressed glioblastoma progression, reducing the spreading area to 0.6 times its original size, indicating a shrinkage of the initial tumor implant.

To assess the treatment’s impact on healthy tissue, cerebral organoid volumes were measured using phase-contrast or bright-field confocal microscopy with manually defined regions of interest. Organoid growth was consistent across all groups, with untreated controls showing a 25% increase in volume over 120 h, and samples treated with TMZ (21%) or **Ir4** (24%) exhibiting similar growth. This indicates that neither **Ir4** nor TMZ caused acute toxicity to healthy organoid tissue.

These findings highlight **Ir4**’s potential as a therapeutic for glioblastoma. It significantly suppresses tumor progression at considerably lower concentrations than TMZ while avoiding acute side effects on healthy tissue. This experimental approach holds promise for developing more effective treatment strategies for this aggressive brain cancer.

## Discussion

Chemotherapy for brain tumors presents significant challenges, primarily due to the difficulty of transporting therapeutic agents across the BBB. While certain chemotherapy drugs are effective against some brain tumors, metal-based antitumor agents remain underutilized, with no metal-based therapies currently approved for clinical use in brain tumors. This study demonstrates that an iridium complex, [Ir­(ttpy)­(C^∧^N)­Cl]­PF_6_, effectively inhibits glioblastoma progression when combined with blue light photoactivation, making it a promising candidate for targeted brain tumor therapy.

All tested iridium complexes (**Ir1**–**Ir10**) exhibited photostability under blue-light irradiation, with **Ir3**–**Ir5** and **Ir10** demonstrating notable photocatalytic activity in generating ROS. Among these, **Ir4**, containing a deprotonated methyl 1-butyl-2-phenylbenzimidazolecarboxylate ligand, showed significant antiproliferative effects following blue light photoactivation in both 2D and 3D glioblastoma models (U87MG) (Table S4). Morphological analysis of treated spheroids, conducted via phase-contrast and confocal microscopy, along with calcein AM-PPI staining, revealed substantial cell death and debris release, indicating cytostatic and cytotoxic effects that inhibit tumor growth.

The ability of **Ir4** to penetrate the BBB was assessed using various *in vitro* models, including a coculture system of U87MG glioblastoma and hCMEC/D3 BBB cells. Results indicated substantial **Ir4** uptake in glioblastoma cells, with lower but significant accumulation in BBB cells. Further investigations confirmed that **Ir4** effectively induced ROS in glioblastoma spheroids following blue laser irradiation, leading to sustained cytotoxic effects.

To more closely approximate *in vivo* conditions, the therapeutic potential of **Ir4** was evaluated in a fusion system comprising cerebral organoids derived from human induced pluripotent stem cells (hiPSC) and glioblastoma spheroids (U87MG). This system enabled real-time monitoring of compound distribution, tumor progression, and the effects of treatment on healthy brain tissue. Results demonstrated the selective accumulation of Ir4 in tumor tissue (Figure 3, panel II), supporting its potential use in photodynamic therapy (PDT) and fluorescence-guided tumor resection.

Long-term studies using an implant model further confirmed reduced tumor progression following **Ir4** treatment and blue light irradiation. However, the presence of residual malignant cells suggests the need for treatment optimization through repeated applications or combination therapies.

While **Ir4**’s activation requires blue-light irradiation (420 nm), which has limited penetration into deep tissues, advancements in laser and fiber-optic technology enable targeted delivery even to deep-seated tumors.^50^ Conversely, longer-wavelength irradiation, though penetrating deeper, may pose risks to underlying healthy tissue,^51^ making blue light particularly suitable for superficial brain tumors.

In light of evolving ethical and regulatory standards, preclinical drug evaluation is shifting away from traditional animal models, as the FDA no longer mandates animal testing before clinical trials.^22^ Given the limitations of animal models in accurately predicting human responses,^52,53^ alternative approaches such as 3D culture systems and organoid-based models provide more reliable platforms for drug screening. In this context, our ongoing research focuses on further exploring the mechanistic effects of Ir complexes in human tumor-derived organoids, with findings to be presented in a future study.

In summary, our findings highlight the potential of **Ir4** as a novel therapeutic agent for glioblastoma treatment, demonstrating its ability to penetrate the BBB, selectively accumulate in tumor tissue, and induce ROS-mediated cytotoxicity. A pertinent objective for future research is to establish a direct correlation between the photophysical, photochemical, and photobiological properties of the investigated iridium complexes and their molecular structures. However, at the current stage of investigation into their anticancer efficacy, such correlations remain speculative. Nonetheless, considering the therapeutic potential of metal-based anticancer agents capable of traversing the BBB, our findings suggest that the lipophilicity of these iridium complexes significantly influences their cellular uptake and permeability characteristics. Specifically, compound **Ir4**, which incorporates an *n*-butyl substituent, exhibits pronounced lipophilic properties. As presented in Table 1, **Ir4** demonstrated an apparent permeability coefficient (*P*_app_) at least an order of magnitude greater than those observed for **Ir3** and **Ir5**. This substantial increase in *P*_app_ indicates a markedly enhanced ability of **Ir4** to penetrate the blood-brain barrier (BBB). These results underscore the critical role of the *n*-butyl group in augmenting the permeability of this class of iridium complexes across the BBB. Further research is warranted to optimize treatment protocols and explore combination strategies to enhance its clinical efficacy in brain cancer therapy.

## Materials and Methods

### Materials

4′-(*p*-Tolyl)-2,2′:6′,2″-terpyridine, 4-(trifluoromethyl)­benzyl bromide, iodomethane, 2-phenylbenzimidazole, 4-chloro-3-nitrobenzoic acid, butylamine, zinc in powder, ammonium formate, 3,4-(methylenedioxy)­benzaldehyde, 2-naphthaldehyde, 1,2-phenylenediamine, 2-thiophenecarboxaldehyde, triethylamine, trifluoroacetic acid, magnesium sulfate, potassium hexafluorophosphate, dimethyl sulfoxide (DMSO), and ethylene glycol were obtained from Sigma-Aldrich (Madrid, Spain). IrCl_3_ was obtained from Johnson Matthey. Deuterated solvents were obtained from Euriso-top. The purity of the synthesized complexes, >95%, used for biological evaluation, was determined by elemental analysis and RP-HPLC. The ^1^H and ^13^C­{^1^H} NMR spectra were recorded on a Bruker AC 300E, Bruker AV 400, or Bruker AV 600 NMR spectrometer, and chemical shifts were determined by reference to the residual ^1^H and ^13^C­{1H} solvent peaks. The C, H, N, and S analyses were performed with a Carlo Erba model EA 1108 microanalyzer, with the EAGER 200 software. Synthesis of complexes was carried out in a 10 mL vial for an Anton Paar Monowave 50 (315 W) microwave. The column was a Zorbax Eclipse Plus C18, 2.1 × 50 mm, 1.8 μ. ESI mass (positive mode) analyses were performed on an RP-/MS TOF 6220. The isotopic distribution of the heaviest set of peaks matched very closely to that calculated for formulating the complex cation in every case.

Human prostate adenocarcinoma derived from the human glioblastoma U87MG cell line was purchased from the American Type Culture Collection (ATCC; USA). For 2D experiments, cells were cultured in DMEM medium (Biosera) supplemented with 10% fetal bovine serum (FBS) (Biosera) and gentamicin (Merck). Cells were cultured in a humidified CO_2_ incubator (5%) and subcultured 2–3 times per week, according to the proliferation of each cell line. Cells cultured under the 3D spheroid-forming conditions were maintained in DMEM-F12 ham medium supplemented with growth and spheroid forming factors: 2% B27 (Thermo Fisher Scientific Inc., MA, USA), epidermal growth factor (EGF; Sigma-Aldrich, Germany, 20 ng mL^–1^), fibroblast growth factor (FGF2; Sigma-Aldrich, Germany, 10 ng mL^–1^) and bovine serum albumin (BSA) (Sigma-Aldrich, Germany, 0.15%). Blood-brain barrier (BBB) cells, hCMEC/D3, were maintained in EGM-2 MV microvascular endothelial cell growth medium-2 bullet kit (Lonza). Human-induced pluripotent stem cells (hiPSCs) were purchased from Gibco and maintained in fully supplemented StemFlex medium (Gibco).

### Synthesis of Intermediate Diamine A

4-Chloro-3-nitrobenzoic acid was dissolved in methanol. Concentrated H_2_SO_4_ (1 mL) was added dropwise to the reaction mixture. The reaction mixture was refluxed overnight (18 h), cooled, and then concentrated under vacuum. The resulting residue was dissolved in ethyl acetate and washed with a saturated solution of sodium bicarbonate. The organic layer was then dried over sodium sulfate and concentrated *in vacuo* to make the product a white solid.

Methyl 4-chloro-3-nitrobenzoate (1 mmol) was dissolved in dichloromethane (10 mL) in a round-bottom flask equipped with a stirrer and nitrogen atmosphere. 4-(Trifluoromethyl)­benzylamine (2 mmol) was added to it at room temperature with constant stirring, followed by the addition of triethylamine (2 mmol). The reaction mixture was stirred at room temperature for 12 h, and the progress of the reaction was monitored by TLC. After complete conversion, the reaction was quenched with water (10 mL), and the product was extracted with dichloromethane (2 × 10 mL). The combined dichloromethane layer was washed with water (10 mL) and brine (10 mL), dried on sodium sulfate, and concentrated under reduced pressure. The crude product was purified by column chromatography using ethyl acetate–hexane (1:5) as eluent to get 3-nitro-4-((4-(trifluoromethyl)­benzyl)­amino)­benzoate in 84% yield. Subsequently, 3-nitro-4-((4-(trifluoromethyl)­benzyl)­amino)­benzoate (1 mmol) was dissolved in methanol (10 mL) in a round-bottom flask equipped with a stirrer and nitrogen atmosphere. Zinc (3 mmol) was added at room temperature with constant stirring, followed by the addition of ammonium formate (2 mmol) in two batches. The reaction mixture was stirred at room temperature for 5 h, and the progress of the reaction was monitored by TLC. After complete conversion, the reaction was filtered to remove zinc and unreacted ammonium formate. The filtrate was concentrated and then dissolved in dichloromethane, and the mixture was stirred for 30 min. The undissolved material was removed by filtration, and dichloromethane was concentrated under reduced pressure. The crude product was purified by column chromatography using ethyl acetate–hexane (1:2) as eluent to get methyl 3-amino-4-((4-(trifluoromethyl)­benzyl)­amino)­benzoate (**A**) in 80% yield (Scheme S1).

### Synthesis of the HC^∧^N Proligand **HL10**

The diamine **A** (see Supporting Information and Scheme S1) was prepared from 4-chloro-3-nitrobenzoic acid with slight modifications of similar procedures.^54,55^ The new ligand, **HL10**, was synthesized from diamine **A** by slightly modifying the literature (Scheme S2) by a condensation reaction.^56^ First, benzaldehyde (1 mmol) and sodium bisulfide (10 mmol) were stirred in water at 100 °C for 1 h. Then, the diamine previously prepared (1 mmol) was dissolved in EtOH and added to the reaction mixture, which was heated overnight at 80 °C. The solid (**HL10**) was filtered and washed with water and hexane.

**HL10**: 71%. ^1^H NMR (600 MHz, DMSO-*d*_6_) δ 8.34 (dd, J = 1.6, 0.6 Hz, 1H, H_b_), 7.89 (dd, J = 8.6, 1.6 Hz, 1H, H_d_), 7.72 (dd, J = 8.7, 1.2 Hz, 2H, H_o_), 7.66 (d, J = 8.4 Hz, 2H, H_l_), 7.60 (dd, J = 8.6, 0.6 Hz, 1H, H_e_), 7.58–7.50 (m, 3H, H_q+p_), 7.19 (d, J = 8.4 Hz, 2H, H_k_), 5.74 (s, 2H, H_i_), 3.88 (s, 3H, H_u_), 2,50 (s, 3H, H_13_). ^13^C­{^1^H} NMR (151 MHz, DMSO-*d*_6_) δ 166.7 (C_t_), 155.4 (C_g_), 142.3 (C_a_), 141.3 (C_j_), 139.2 (C_f_), 130.4 (C_q_), 129.4 (C_h_), 129.1 (C_o_), 129.0 (C_p_), 126.9 (C_k_), 125.8 (C_l_), 124.2 (C_c_), 124.0 (C_d_), 121.0 (C_b_), 111.3 (C_e_), 52.2 (C_u_), 47.4 (C_i_). Mass ESI-MS (pos. ion mode, DMSO): calc.: [M + H]^+^ = 411.1320 *m*/*z*; exp.: 411.1332 *m*/*z*. Anal. Calc. for C_23_H_17_F_3_N_2_O_2_: %C, 67.31; %H, 4.18; %N, 6.83. Found: %C, 67.32; %H, 4.39; %N, 6.71.

### Synthesis of the Iridium Complex **Ir10**

The new complex I**r10** was prepared following the reported method (Scheme S3).^14^ An orange solid was obtained with a good yield.

**Ir10**: 62%. ^1^H NMR (600 MHz, DMSO-*d*_6_) δ 9.85 (s, 1H, H_b_), 9.27 (s, 2H, H_7_), 8.99 (d, J = 8.0 Hz, 2H, H_4_), 8.28 (d, J = 8.2 Hz, 2H, H_10_), 8.24 (td, J = 8.0, 1.2 Hz, 2H, H_3_), 8.18 (s, 2H, H_e+d_), 7.98 (dd, J = 5.7, 1.2 Hz, 2H, H_1_), 7.76 (d, J = 8.5 Hz, 2H, H_l_), 7.63 (dd, J = 7.5, 0.9 Hz, 1H, H_s_), 7.59–7.53 (m, 6H, H_k+11+2_), 6.82 (ddd, J = 8.0, 7.5, 1.0 Hz, 1H, H_r_), 6.74 (ddd, J = 8.0, 7.7, 0.9 Hz, 1H, H_q_), 6.43 (s, 2H, H_i_), 6.15 (dd, J = 7.7, 1.0 Hz, 1H, H_p_), 3.87 (s, 3H, H_u_), 2.50 (s, 3H, H_13_). ^13^C­{^1^H} NMR (151 MHz, DMSO-*d*_6_) δ 166.3 (C_t_), 163.3 (C_g_), 158.0 (C_5_), 155.6 (C_6_), 152.6 (C_1_), 151.3 (C_8_), 143.5 (C_o_), 141.4 (C_12_), 140.6 (C_a_), 140.4 (C_j_), 140.0 (C_3_), 139.7 (C_f_), 132.8 (C_h_), 132.3 (C_9_), 131.1 (C_p_), 131.0 (C_q_), 129.9 (C_11_), 129.0 (C_2_), 128.5, 128.3, 128.1 (C_10_), 127.0 (C_k_), 126.2 (C_s_), 126.1 (C_c_), 125.9 (C_l+4_), 125.2 (C_d_), 125.0, 123.8 (C_r_), 123.2, 121.2 (C_7_), 120.4 (C_b_), 111.7 (C_e_), 52.3 (C_u_), 47.6 (C_i_), 21.0 (C_13_). Mass ESI-MS (pos. ion mode, DMSO): calc.: [M–PF_6_]^+^ = 960.1904 *m*/*z*; exp.: 960.1878 *m*/*z*. Anal. Calc. for C_45_H_33_ClF_9_IrN_5_O_2_P: %C, 48.90; %H, 3.01; %N, 6.34. Found: %C, 49.19; %H, 3.27; %N, 6.32.

### Photophysical Characterization of the Compounds

UV/vis spectroscopy was performed on a PerkinElmer Lambda 750 S spectrometer with the operating software. The solution of the complex was prepared in acetonitrile and water (1% DMSO) at 10 μM. Emission spectra were obtained with a Horiba Jobin Yvon Fluorolog 3–22 modular spectrofluorometer with a 450 W xenon lamp. Measurements were performed in a right-angled configuration using 10 mm quartz fluorescence cells for solutions at 298 K. Emission lifetimes (τ) were measured using an IBH FluoroHub TCSPC controller and a NanoLED (372 nm) pulse diode excitation source (τ < 10 μs); the estimated uncertainty is ±10% or better. Emission quantum yields (Φ) were measured using a Hamamatsu C11347 absolute PL quantum yield spectrometer, with an estimated uncertainty of ±10% or better. The solution of the new complex **Ir10** was prepared in acetonitrile at 10 μM. For lifetimes and quantum yield measurements, the sample was previously degassed by bubbling argon for 20 min.

### Stability and Photostability of Iridium Complexes **Ir1**–**Ir10**

The stability of complex **Ir10** was studied in DMSO, water (1% DMSO), and RPMI (5% DMSO) using UV/vis spectroscopy at different times in the absence and presence of GSH (10 mM) or NADH (100 μM) to mimic the cellular and physiological conditions (Figure S6).

The photostability of complexes **Ir1**–**Ir10** in DMSO was tested by UV/vis spectra at *t* = 0 and after 2 h of irradiation with a blue light photoreactor (EXPO-Panels from Luzchem (Canadá)) (465 nm, 5.0 mW/cm^2^).

### Antiproliferative Activity

The antiproliferative activity of the compounds under investigation was assessed using the Sulforhodamine B (SRB) assay on both 2D cell monolayers and 3D spheroids. For the 2D cell monolayer assay, cells were seeded in 96-well tissue culture plates at a density of 3 × 10^3^ cells/well in 100 μL of culture medium. Following overnight incubation in a humidified CO_2_ incubator, the medium was replaced, and the cells were treated with the investigated compounds in a final volume of 200 μL/well of Earle’s Balanced Salt Solution (EBSS). The cells were then exposed to the tested compounds for 1.5 h under dark conditions (5% CO_2_, 37 °C) and subsequently irradiated with blue light (420 nm) for 30 min at an intensity of 58 W/m^2^. After treatment, the samples were washed with Phosphate-Buffered Saline (PBS) and replenished with 200 μL of culture medium. Following an additional 70-h incubation, the cells were washed with PBS, fixed with trichloroacetic acid at 4 °C for 1 h, and stained with 0.4% SRB dye for 30 min. The stained cells were dissolved with Tris (10 mM), and the cell viability was assessed by measuring the absorbance at 570 nm using an Absorbance Reader (SPARK TECAN, SCHOELLER). The absorbance readings were then converted to the percentage of control (% cell survival), and the antiproliferative effects were expressed as IC_50_ values, which were calculated from curves plotting cell survival (%) versus drug concentration (μM) (IC_50_ = the concentration of the agent that inhibits cell growth by 50%). For experiments with 3D spheroids, 2500 single cells/well were seeded on ultralow attachment U-shape 96-well plates (Corning, USA) and cultured for 72 h in 3D forming medium DMEM-F12 ham medium supplemented with growth and spheroid forming factors: 2% B27 (Thermo Fisher Scientific Inc., MA, USA), epidermal growth factor (EGF; Sigma-Aldrich, Germany, 20 ng mL^–1^), fibroblast growth factor (FGF2; Sigma-Aldrich, Germany, 10 ng mL^–1^), and BSA (0.15%). After the 72-h spheroid formation period, the samples were treated and irradiated as described for the 2D cultures. Subsequently, the samples were cultured in the 3D forming medium for an additional 70 h, and the antiproliferative potency of the tested compounds against spheroids was determined by assessing changes in IC_50_ values using the CellTiterGlo-3D assay (Promega). The luminescence signal was detected using the plate reader SPARK (Tecan, Manedorf, Switzerland), and the IC_50_ values were calculated from the survival curves. All experiments were conducted in triplicate to ensure the reliability and reproducibility of the results. Furthermore, the concentrations of the compounds in the medium during the treatment were verified using Flame Atomic Absorption Spectroscopy (FAAS) or Inductively Coupled Plasma Mass Spectrometry (ICP-MS).

### Blood-Brain Barrier Penetration of Tested Compounds

The BBB model was established utilizing hCMEC/D3 cells, acknowledged as the most reliable cells suitable for *in vitro* screening of drug penetration through the BBB.^36,38,57^ The hCMEC/D3 cells were cultured on Collagen I-coated tissue culture plastics in the EGM-2 MV microvascular endothelial cell growth medium-2 supplemented with hEGF, VEGF, R3-IGF-1, ascorbic acid, hydrocortisone, hFGF-β, FBS, and a gentamicin/amphotericin mixture. To establish the BBB, hCMEC/D3 cells were seeded at a density of 2 × 10^4^ cells per 0.33 cm^2^ culture insert with a 3 μm pore size (Falcon). Before cell seeding, transwell inserts were coated with collagen I. Cells were cultured for an additional 7–10 days to establish an effective barrier. The barrier function was assessed by measuring the trans-epithelial electrical resistance (TEER), and treatment with **Ir3**, **Ir4**, and **Ir5** at a concentration of 10 μM was initiated when the control wells reached a TEER of approximately 60–80 Ω·cm^2^. The concentrations of the tested iridium compounds were analyzed using inductively coupled plasma mass spectrometry (ICP-MS), and the apparent permeability coefficients (*P*_app_) were calculated using the formula *P*_app_ = (d*C*/d*t*)·(*V*_r_/*AC*_0_), where d*C*/d*t* represents the cumulative concentration of the corresponding iridium compound over time in the basolateral compartment, *V*_r_ is the volume (1.5 mL), *A* is the surface insert area (0.336 cm^2^), and *C*_0_ is the initial concentration of the compound in the apical part.

### Co-Cultivation Model of BBB with U87MG Cells with Subsequent Cellular Uptake Analysis

To confirm that the tested compounds can effectively target and accumulate in the desired U87MG glioblastoma cells, a coculture model was employed to monitor cellular uptake in both hCMEC/D3 cells and U87MG cells. U87MG glioblastoma cells were seeded in the basolateral parts of 24-well plates at a density of 1 × 10^5^ cells per well and cultured overnight. Cell culture inserts containing a preformed BBB constructed of hCMEC/D3 cells were then added to the apical part of the model system. Following the BBB preformation phase (refer to the preceding paragraph for detailed information), the apical parts were treated with 10 μM of **Ir4**. Subsequently, the apical and basolateral parts were assembled in the 24-well plate format. The total iridium uptake by U87MG or hCMEC/D3 cells was analyzed 24 h after treatment. In brief, the apical and basolateral parts were separated, and each cellular model was harvested by trypsinization, pelleted by centrifugation, and counted. After obtaining the final pellet, the samples were treated with ultrapure HCl (5 M) and mineralized for at least 24 h. The final iridium content was determined through ICP-MS analysis. The experiments were replicated in triplicate, and the data are expressed as nanograms of iridium per one million cells.

### Distribution of Tested Iridium Compounds in Matrigel-Embedded U87MG Spheroids by Confocal Microscopy

Spheroids derived from U87MG cells were encapsulated in a Matrigel matrix. The U87MG cells were incubated for 72 h under 3D conditions using ULA 96-well U-shaped plates (Corning) and cultured in DMEM-F12 ham medium supplemented with growth factors conducive to spheroid formation: 2% B27 (Thermo Fisher Scientific Inc., MA, USA), epidermal growth factor (EGF; Sigma-Aldrich, Germany, 20 ng mL^–1^), fibroblast growth factor (FGF2; Sigma-Aldrich, Germany, 10 ng mL^–1^), and BSA (0.15%). Following the 72-h spheroid formation phase, the samples were embedded in a Matrigel matrix and allowed to gel completely (30 min). Afterward, the spheroids were further cultured for 24 h in confocal Petri dishes (Mattek) with 3D forming medium. Subsequently, the samples were exposed to 5 μM of the tested compounds 3, 4, and 5 for 3 h. The samples were then rinsed, transferred to a fresh 3D forming medium, and visualized using a confocal microscope (Leica CM SP8 SMD, Leica, Germany). Imaging was conducted in defined *Z*-stacks with a 20 μm step, and fluorescence intensities were captured and analyzed using ImageJ software.

### Precise Single-spheroid Irradiation by Confocal Microscopy with Subsequent Calcein AM-Propidium Iodide Staining

Matrigel-embedded spheroids were treated for 3 h with the investigated compounds at concentrations corresponding to the IC_50_ determined for 3D cultures. Then, the samples were irradiated for 60 s with 405 nm laser light (1 mW) and placed in a drug-free, 3D forming medium for an additional 21 h. Then, treated and irradiated (or dark incubated controls) were stained with Hoechst 33258 (20 μg mL^–1^), Calcein AM (2 μM), and PPI (8 μg mL^–1^) for 2 h. Samples were sequentially imaged on a confocal microscope Leica CM SP5 (Leica, Germany), in 10 *z*-stack scans (20 μm each). Images were processed using ImageJ software. Fluorescence intensities were assessed for the maximal projections of the *z*-stacks, and the corresponding Calcein AM/PPI ratio was calculated. Hoechst dye was used to accurately define the Region of Interest (ROI) mask and analyze fluorescence intensities. Three independent experiments were carried out to ensure the reliability and reproducibility of the results. The representative images are shown.

### Detection of ROS in U87MG Spheroids by Confocal Microscopy

The spheroids were exposed to **Ir4** (IC_50_, U87MG 3D, 72 h) for 3 h or maintained under drug-free conditions (control). Subsequently, the samples were stained with CellRox reagent (5 μM) and PPI (8 μg mL^–1^) before being irradiated for 60 s with blue laser light (405 nm, 1 mW). Imaging of the samples was conducted before or immediately after the irradiation (1 min postirradiation) and at defined time intervals (15 and 30 min). The fluorescence scanning was performed sequentially in *z*-stack mode (10 *z*-stacks). Postirradiation imaging was conducted for a total of 90 min, with only the data collected up to 30 min being presented and quantified. The experiments were independently replicated in triplicate.

### Generation of Cerebral Organoids and Organoid-spheroid Implant Model for Distribution Studies

An implant fusion model was established to monitor the distribution of **Ir4** within the tumor, represented by the implanted tumorspheres generated from U87MG, and healthy tissue, represented by the hiPSC-derived cerebral organoid. Before the implantation procedure, spheroids derived from U87MG glioblastoma were cultured for 72 h under 3D forming conditions using ULA plates (96-well U-shaped plate, Corning) in DMEM-F12 supplemented with 2% B27, epidermal growth factor (EGF) at 20 ng mL^–1^, fibroblast growth factor 2 (FGF2) at 10 ng mL^–1^, and BSA (0.15%). Cerebral organoids were generated through a four-step directed differentiation process from human induced pluripotent stem cells (hiPSCs, Gibco). The hiPSCs were cultured on Geltrex matrix (hESC-Qualified, Reduced Growth Factor Basement Membrane Matrix; Gibco) in StemFlex basal medium (DMEM/F12, Ham, 1:1) fully supplemented with StemFlex supplement (Gibco). The preformation of cerebral organoids, involving the formation of embryoid bodies (EBs), was maintained in U-shape ultralow attachment 96-well plates (ULA, Corning) with 9000 hiPSCs per well in fully supplemented Stemdiff Cerebral organoid medium and 10 μM of the rock inhibitor Y27632. The EBs were cultured for 4 days to establish round-shaped EBs with a diameter of approximately 400 μm. Subsequently, 2 EBs per well were transferred to 24-well ULA plates, and differentiation into neural ectoderm was induced for an additional 2 days using the Stemdiff Cerebral Organoid Kit (StemCell Technologies, CA). Following this, early organoids were embedded in the Matrigel matrix (suitable for organoid culture, Corning) and directed toward further differentiation into neuroectoderm. After the initial differentiation phase, early organoids were monitored for the development of optically clear neuroectoderm (characterized by an optically bright, translucent surface) and the formation of large buds of translucent ectoderm that were not radially organized. Postembedding, the organoids were cultured on 6-well ULA plates under continuous orbital shaker agitation (50 rpm). The organoids were cultured for an additional 25 days to develop fully matured cerebral organoids with a mean diameter of 1.5–2.5 mm. These organoids served as a model system for healthy brain tissue. The preparation protocol for cerebral organoids represents a robust and well-established tool adopted from the work of Lancaster et al.^58^ After a designated period of spheroid growth (72 h) and complete maturation of the cerebral organoids (approximately 35 days), the fusion implant system was prepared. Organoids were stripped of the Matrigel capsule using CellRecovery solution (Corning). Subsequently, spheroids and Matrigel-free organoids were placed on the silicon organoid embedding sheet (Stemcell Technologies). Following this, both entities were manually positioned close to each other and coembedded into the Matrigel capsule (Corning). After complete gelling, each implant was examined under a phase-contrast microscope, and the implant model was cultured for 24 h in an organoid maturation medium. Subsequently, the DU145+ organoid and U87MG+ organoid fusion systems were treated with 5 μM of **Ir4** and incubated for 24 h to facilitate the complete distribution of the tested compound within the healthy and malignant tissues. The samples were then transferred to confocal Petri dishes containing drug-free cerebral maturation medium and analyzed using a confocal microscope (Leica CM SP5, Leica, Germany).

### The Effects of Treatment with **Ir4** Combined with Blue Laser Light Irradiation on a Cerebral Organoid-Glioblastoma Implant Model

The implant model was prepared as described in the preceding paragraph. After 24 h postimplantation, the U87MG-organoid system was exposed to **Ir4** (concentration corresponding to IC_50_, U87MG 3D, 72 h) for 3 h. Subsequently, the specific area of implantation where the U87MG spheroid was placed underwent selective irradiation with blue laser light (405 nm, 60 s, 1 mW) using the Leica CM SP5 confocal microscope (Leica, Germany). The system was then maintained under drug-free conditions for an additional 120 h. After the designated period, the samples were stained with Hoechst 33258 (20 μg mL^–1^), Calcein AM (2 μM), and PPI (8 μg mL^–1^) for 2 h. The samples were then sequentially imaged using the Leica CM SP5 confocal microscope in the *z*-stacking mode.

## Supplementary Material



## Notes

HPLC spectra; UV–vis spectra of complexes **Ir1**–**Ir10** for photostability studies; UV–vis spectra for photocatalytic oxidation of NADH studies; UV–vis spectra for photocatalytic singlet oxygen and hydroxy radical photogeneration studies (PDF)

## Abbreviations

3D: three-dimensional
ACQ: aggregation-caused quenching
AIE: aggregation-induced emission
BBB: blood-brain barrier
DMSO: dimethyl sulfoxide
DPBF: 1,3-diphenylbenzofuran
ECM: extracellular matrix
ETC: electron transport chain
hiPSC: human induced pluripotent stem cell
HPF: hydroxyphenyl fluorescein
IC_50_: concentration of the compound reducing cell growth by 50%
ICP-MS: inductively coupled plasma mass spectrometry
ISC: intersystem crossing
LLCT: ligand-to-ligand charge transfer
MLCT: metal-to-ligand charge transfer
MTT: 3-[4,5-dimethylthiazol-2-yl]-2,5 diphenyl tetrazolium bromide
NADH: nicotinamide adenine dinucleotide;
*P*_app_: apparent permeability
PDT: photodynamic therapy
PPI: propidium iodide
RFP: red fluorescent protein
ROI: region of interest (ROI)
ROS: reactive oxygen species
SD: standard deviation
TEER: trans-endothelial electrical resistance (TEER)
TMI: tumor mass index
TMZ: Temozolomide TMZ
TOF: turnover frequency
