# Supplementary material for: Photoactivatable Cyclometalated Ir(III) Compound Penetrates the Blood-Brain Barrier in 3D Spheroidal and Advanced 3D Organoid Models of Inherently Resistant and Aggressive Brain Tumors
Source: ACS Pharmacol Transl Sci. 2025 Jun 30;8(7):2033–47. doi: 10.1021/acsptsci.5c00145 (PMC12261233; doi:10.1021/acsptsci.5c00145)
Supplement: Supplementary file 1 [file pt5c00145_si_001.pdf]

Supplementary Information

Photoactivatable Cyclometalated Ir(III) Compound Penetrates the Blood-Brain Barrier in 3D Spheroidal and Advanced 3D Organoid Models of Inherently Resistant and Aggressive Brain Tumors

Vojtech Novohradsky[a], Alicia Marco[b], Marie Svitelova[a], Natalia Cutillas[b], José Ruiz[b,*], Viktor Brabec[a,c,*]

[a] *Czech Academy of Sciences, Institute of Biophysics, Kralovopolska 135, CZ-61200 Brno, Czech Republic*
[b] *Departamento de Química Inorgánica, Universidad de Murcia and Institute for Bio-Health Research of Murcia (IMIB-Arrixaca), Murcia E-30100, Spain*
[c] *Department of Biophysics, Faculty of Science, Palacky University, Slechtitelu 27, 779 00 Olomouc, Czech Republic*

*Corresponding Authors:
José Ruiz (jruiz@um.es) and Viktor Brabec (vbrabec44@gmail.com)

TABLE OF CONTENTS

Photocatalytic properties of Ir1–Ir10 in cell-free media. S-3

Photooxidation of NADH. S-3

Quantum yields for the generation of $^1O_2$. S-3

Photocatalytic generation of ·OH in cell-free media. S-3

Tables S-4

Table S1. Excitation (λ_{exc}) and emission (λ_{em}) wavelengths, emission lifetime (τ_{em}), and emission quantum yield (Φ_{em}) of complexes **Ir10** and **Ir4** in deaerated acetonitrile**.** S-4

Table S2. TON and TOF (h^{-1}) values calculated for complexes **Ir3–Ir5** and **Ir10** after blue light irradiation S-4

Table S3. Quantum yield of $^1O_2$ generation of complexes **Ir3–Ir5** and **Ir10** under blue light irradiation. S-4

Table S4. The antiproliferative activity of the investigated compounds determined in 2D and 3D models of U87MG cells S-5

Figures S-6

Nuclear magnetic resonance (NMR) spectra (**Figures S1-S13**). S-6

High-resolution mass spectra (**Figures S14-S15**). S-13

HPLC chromatograms (**Figures S16-S17**). S-15

UV/Vis and emission (λ_{exc} = 405 nm) spectra of complex **Ir10** (10 µM) in aerated (A) acetonitrile and (B) water (1% DMSO) (**Figure S18**). S-17

Time dependence of the UV/Vis spectrum of complex **Ir10** in the dark in (A) DMSO, (B) RPMI (5% DMSO), and (C) water (1% DMSO) in the absence and presence of GSH or NADH (**Figures S19**). S-18

Emission spectra of complexes **Ir4** and **Ir10** in different water/DMSO mixtures (**Figures S20-S21**). S-19

Photostability of the complexes **Ir1–Ir10** in DMSO by UV/Vis after 2 h of blue light irradiation (**Figure S22**). S-20

UV/Vis spectra for the photocatalytic oxidation of NADH by iridium complexes in PBS (5% DMSO) under blue light irradiation at r.t. (**Figure S23**). S-22

NADH negative control assays (**Figures S24-S25**). S-23

Evolution of the absorption spectra of DPBF in the presence of complexes **Ir3–Ir5**, **Ir10** and $[Ru(bpy)_3]^{2+}$ in acetonitrile after blue light irradiation (**Figures S26-S28**). S-24

Evolution of the emission spectra of HPF (10 µM) in the presence of complexes **Ir3–Ir5 or Ir10** (10 µM) in PBS (5% DMF) under blue light irradiation (**Figure S29**). S-25

Analysis of the morphology of the brain glioblastoma U87MG spheroids treated with the investigated iridium complexes **Ir1** – **Ir10** and temozolomide (TMZ) (**Figure S30**). S-26

Example of organoid-spheroid implants immediately after implantation and 24 h post-implantation (**Figure S31**). S-27

4. Schemes S-28

Synthesis of intermediate diamine **A (Scheme S1)**. S-28

Synthesis of the HC^N proligand **HL10 (Scheme S2)**. S-28

Synthesis of the iridium complex **Ir10 (Scheme S3)**. S-28

References. S-29

1. Photocatalytic properties of complexes Ir1–Ir10 in cell-free media.

1.1. Photooxidation of NADH.

The ability of the complexes to photocatalyse the oxidation of NADH was evaluated by recording the absorption spectra of NADH (100 µM) in the presence of complex (5 µM) in PBS (5% DMF) upon blue light irradiation (EXPO-Panels from Luzchem (Canadá)) (465 nm, 5.0 mW/cm^2).[1]

1.2. Quantum yields for the generation of $^1O_2$.

Singlet oxygen is considered the primary cytotoxic species in Type II PDT processes by transferring energy.[1, 2] To assess the ability of complexes **Ir3–Ir5** and **Ir10** to generate singlet oxygen ($^1O_2$) under photocatalytic conditions, singlet oxygen production was evaluated spectroscopically in acetonitrile by the decreasing of 1,3-diphenylbenzofuran (DPBF) absorbance at 411 nm (Figure S11) upon irradiation with blue light (λ = 465 nm, 0.7 mW/cm^2) in the presence of complexes **Ir3–Ir5** and **Ir10**. To obtain singlet oxygen quantum yields, $[Ru(bpy)_3]Cl_2$ was used as a reference (0.57; Figure S12). The quantum yield was calculated according to the following equation:

$$\Phi_{\Delta S} = \Phi_{\Delta R}\left(\frac{m_S}{m_R}\right)\left(\frac{1-10^{AR}}{1-10^{AS}}\right)$$

Where $\Phi_{\Delta R}$ is the reference quantum yield, m_R and m_S are the slopes of the reference and sample, respectively, and A_R and A_S are the absorbance of the reference and the sample at the irradiation wavelength.

1.3. Photocatalytic generation of ·OH in cell-free media.

We also investigated the complexes' ability to generate hydroxyl radicals (•OH), a specific type-I ROS, in PBS (5% DMF) by using a spectroscopic method based on the oxidation of the nonfluorescent hydroxyphenyl fluorescein (HPF) probe by OH· to the corresponding fluorescent product.[3] The emission of HPF (10 µM) was measured at different times in the presence of complex (10 µM) in PBS (5% DMF) under blue light irradiation (EXPO-Panels from Luzchem (Canada).

2. Tables

Table S1. Excitation (λ_{exc}) and emission (λ_{em}) wavelengths, emission lifetime (τ_{em}), and emission quantum yield (Φ_{em}) of selected iridium complexes in deaerated acetonitrile.

Complex	λ_{exc}[a] (nm)	λ_{em} (nm)	τ_{em}[b] (μs)	Φ_{em}[b] (%)
**Ir10**	390	550	2.71	64.5
**Ir1**[c]	350	580	0.34	9.4
**Ir3**[c]	350	560	2.16	65.1
**Ir4**[c]	370	555	2.63	72.1
**Ir5**[c]	370	545	0.73	20.9

[a] λ_{exc} maxima. [b] Emission lifetimes ($\lambda_{NanoLED}$= 372 nm) and quantum yields measured in deaerated solution. [c]Data for **Ir1**, **Ir3, Ir4,** and **Ir5** taken from reference [*J. Med. Chem.* **2023,** *66,* 9766-9783].

Table S2. TON and TOF (h^{-1}) values calculated for catalysts **Ir3–Ir5** and **Ir10** for NADH photo-oxidation after blue light irradiation (λ = 465 nm, 4.8 mW/cm^2).

Complex	Ir3	Ir4	Ir5	Ir10
**TON**	11.32	10.25	13.38	14.01
**TOF (h^{-1})**	113.18	38.43	20.07	28.02

Table S3. Quantum yield of $^1O_2$ generation of complexes **Ir3–Ir5** and **Ir10** under blue light irradiation (λ = 465 nm, 0.7 mW/cm^2).

Complex	Ir3	Ir4	Ir5	Ir10
**Φ_Δ**	0.95	0.87	0.58	0.88

Table S4

The antiproliferative activity (IC_{50} values[a]) of the investigated compounds determined in 2D and 3D models of U87MG cells[b]

	**2D**[c]			**3D**[d]			TMI[f]
	Dark	Irradiated	PI[d]	Dark	Irradiated	PI[e]	(3D/2D)
**Ir1**	4.3 ± 0.1	1.3 ± 0.1	3.5	16.4 ± 0.2	5.7 ± 0.7	2.9	4.4
**Ir2**	6.9 ± 0.4	0.43 ± 0.02	16	14.9 ± 0.5	5.0 ± 0.2	3.0	11.6
**Ir3**	30.4 ± 4.3	0.67 ± 0.08	45	36.6 ± 4.5	6.0 ± 1.0	6.1	9.0
**Ir4**	21.3 ± 1.4	0.52 ± 0.08	41	27.6 ± 4.0	2.1 ± 0.2	13.1	4.0
**Ir5**	25.3 ± 2.1	0.42 ± 0.03	60	32.3 ± 3.3	7.1 ± 0.8	4.5	16.9
**Ir6**	23.9 ± 3.1	1.0 ± 0.1	23	26.0 ± 5.1	8.3 ± 1.1	3.1	8.3
**Ir7**	7.1 ± 0.4	1.5 ± 0.2	4.7	10.8 ± 1.6	6.7 ± 0.6	1.6	4.5
**Ir8**	9.3 ± 0.9	0.80 ± 0.05	12	19.3 ± 0.7	7.0 ± 0.9	2.8	8.8
**Ir9**	6.2 ± 0.7	1.3 ± 0.2	4.6	18.9 ± 0.2	7.3 ± 0.3	2.6	5.6
**Ir10**	34.2 ± 5.3	2.2 ± 0.9	15.5	41.3 ± 1.6	10.5 ± 0.6	3.9	4.8
Temozolomide	≥100	-	-	≥100	95.3 ± 6.4	≥1.2	

[a] Concentration that causes 50% inhibition of cell proliferation. [b] Cell monolayer or generated 3D spheroids were treated for 90 min with increasing concentrations of the investigated compounds, followed by 30 min of irradiation with blue light (420 nm; 58 Wm^{-2}) and 70 h of drug-free incubation. [c] IC_{50} values were determined by SRB assay. [d] IC_{50} values were determined by CellTiter-Glo® 3D chemiluminescent assay. [e] PI - phototoxicity index was calculated as the ratio of IC_{50} determined under the dark conditions / IC_{50} determined for 420 nm irradiated samples.

[f] TMI – tissue-mass index was calculated as the ratio of IC_{50} values determined for 3D and 2D models under irradiation conditions.

3. Figures.

Nuclear magnetic resonance (NMR) spectra.

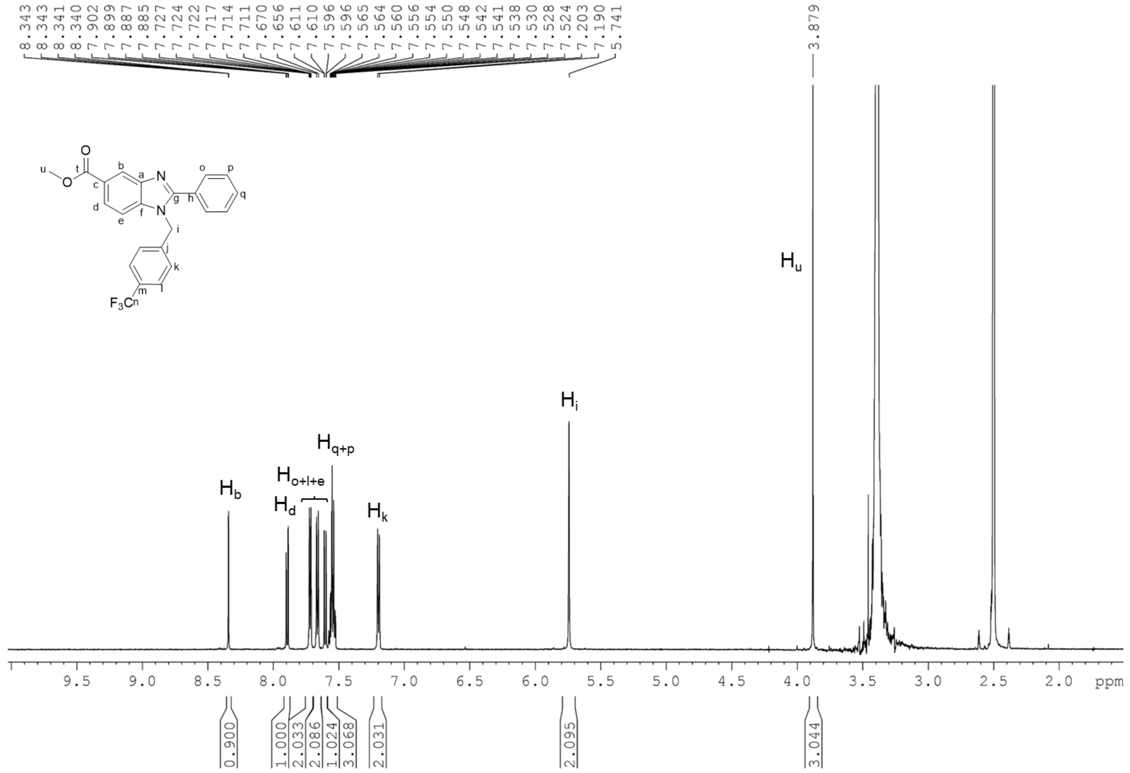

Figure S1. ^{1}H NMR spectra of **HL10**, 600 MHz, DMSO-d_6.

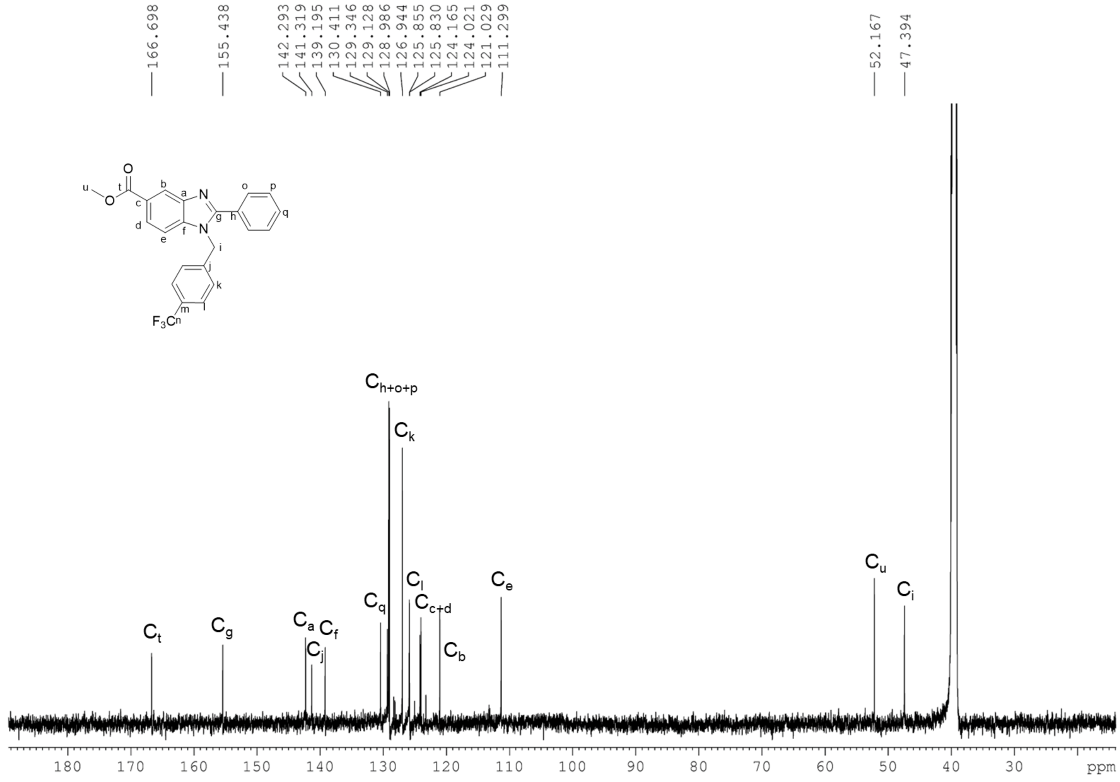

Figure S2. ^{13}C NMR spectra of **HL10**, 151 MHz, DMSO-d_6.

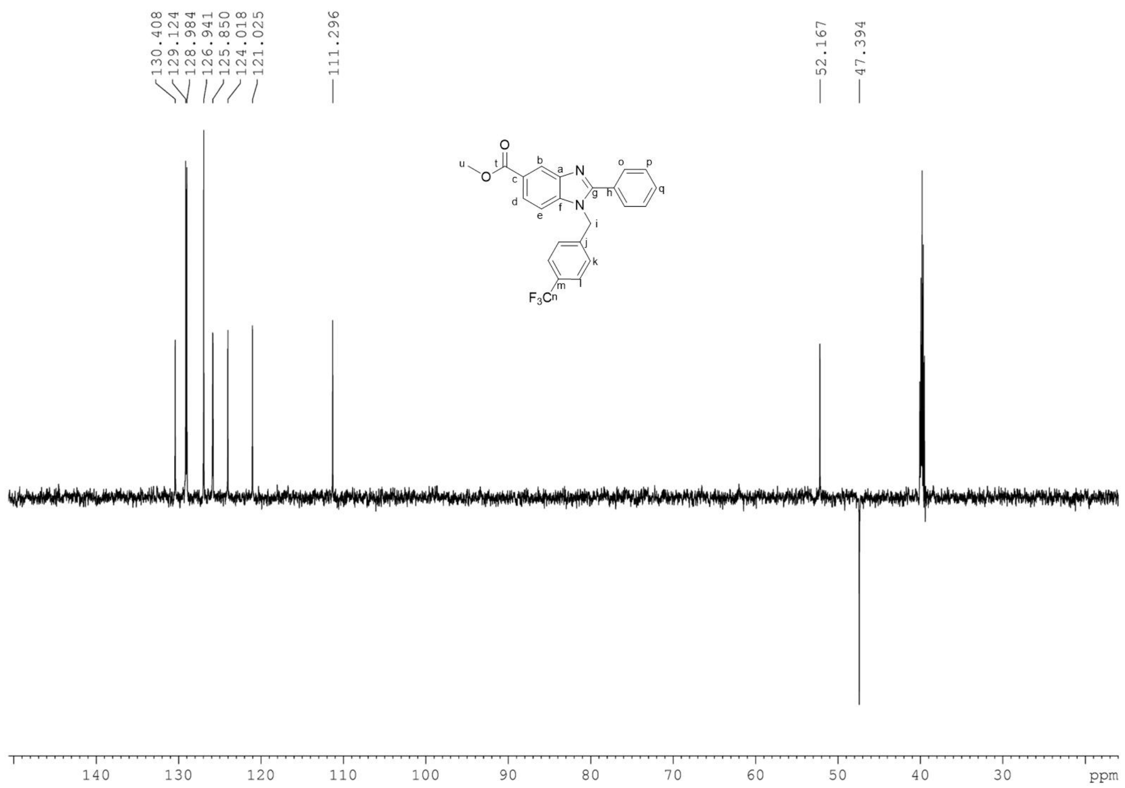

Figure S3. DEPT-135 NMR spectrum of **HL10**, 151 MHz, DMSO-d_6.

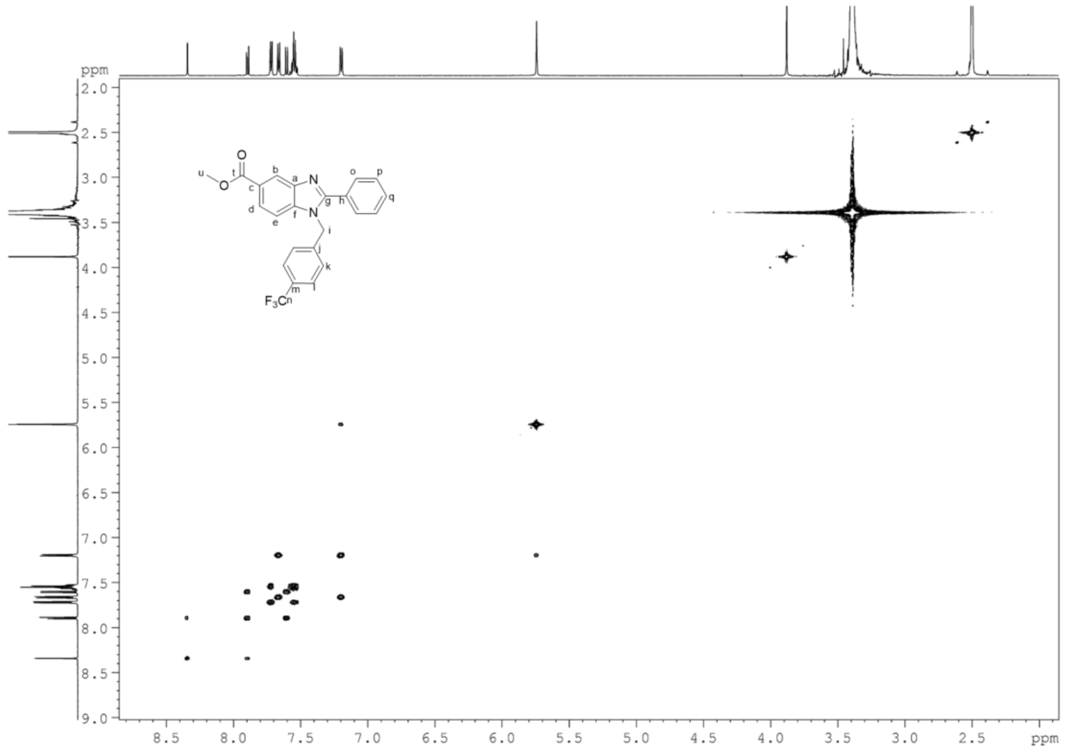

Figure S4. ^1H-^1H COSY NMR spectrum of **HL10**, 600 MHz, DMSO-d_6.

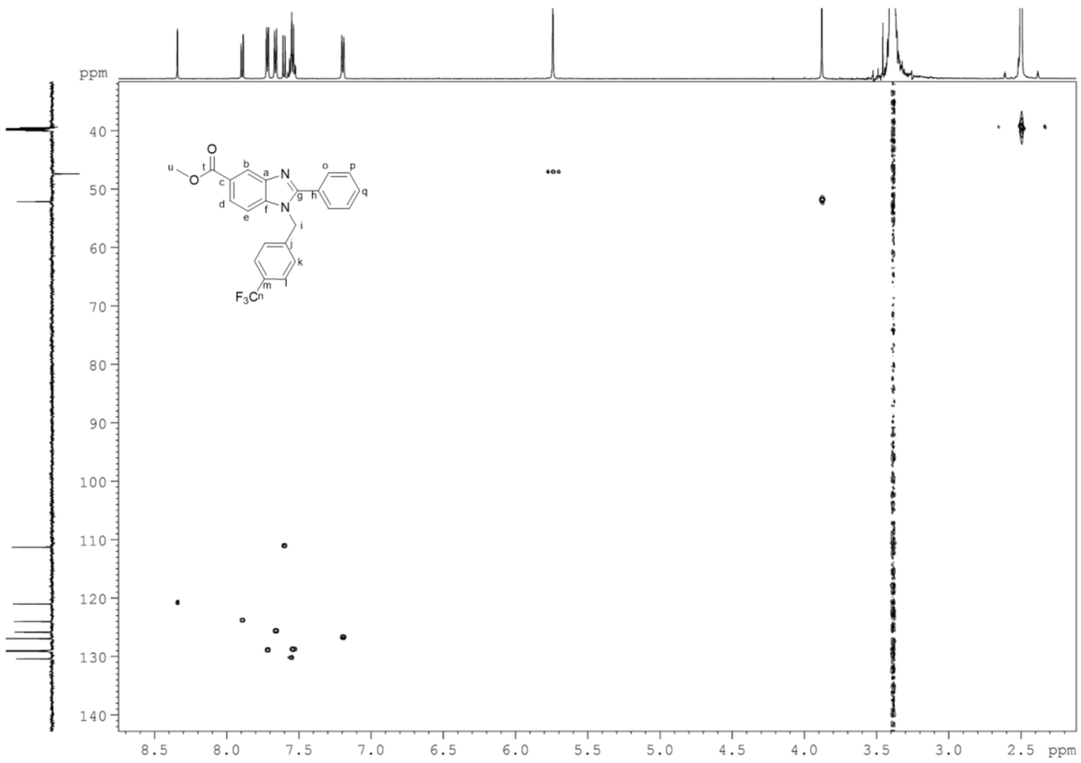

Figure S5. ^{1}H-^{13}C HSQC NMR spectrum of **HL10**, 600 MHz, DMSO-d_6.

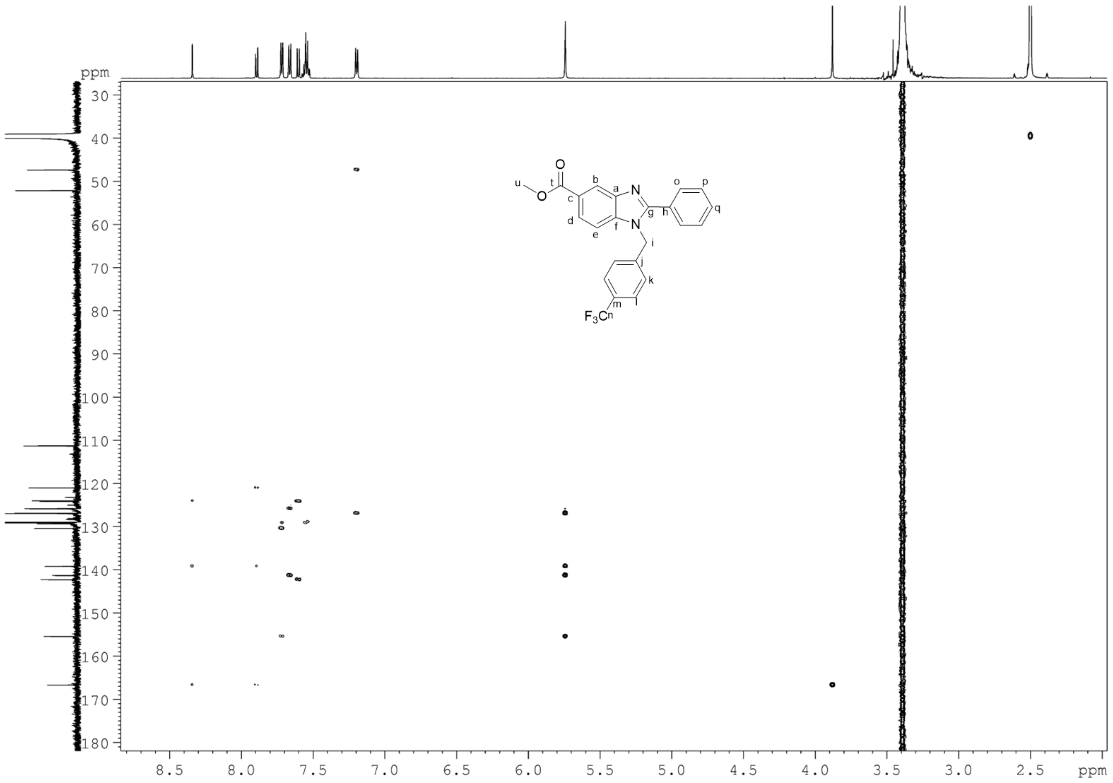

Figure S6. ^{1}H-^{13}C HMBC NMR spectrum of **HL10**, 600 MHz, DMSO-d_6.

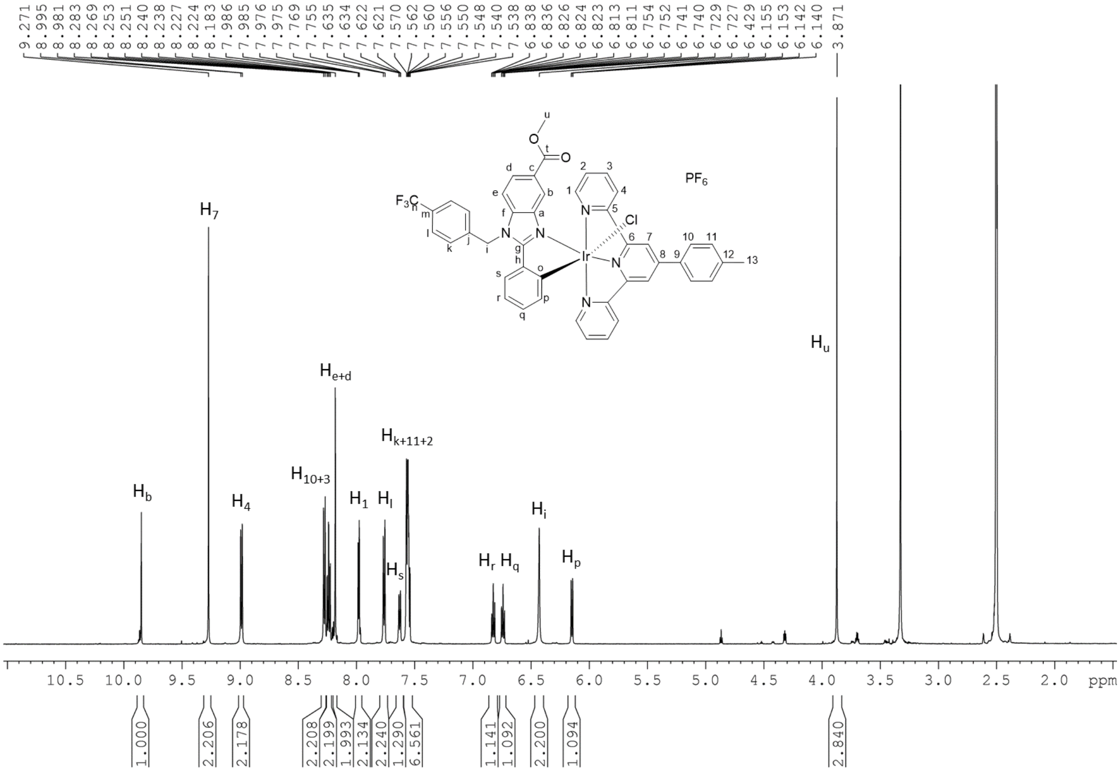

Figure S7. ^{1}H NMR spectra of **Ir10**, 600 MHz, DMSO-d_6.

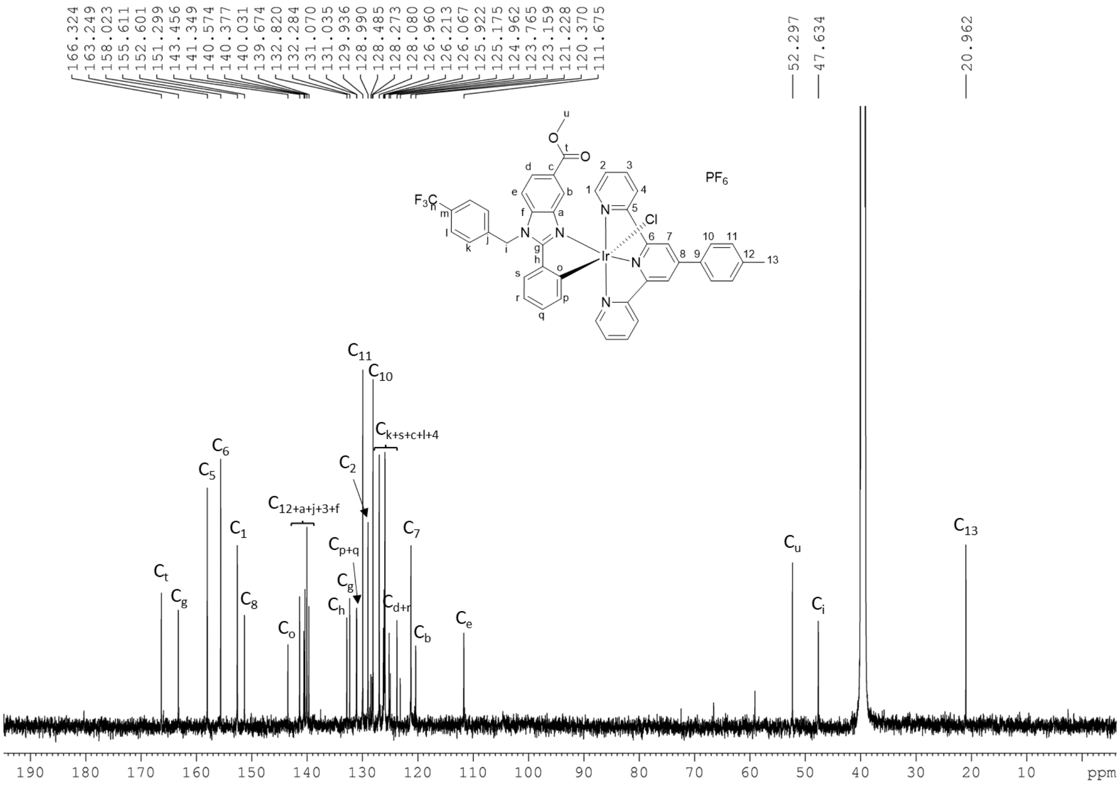

Figure S8. ^{13}C NMR spectra of **Ir10**, 151 MHz, DMSO-d_6.

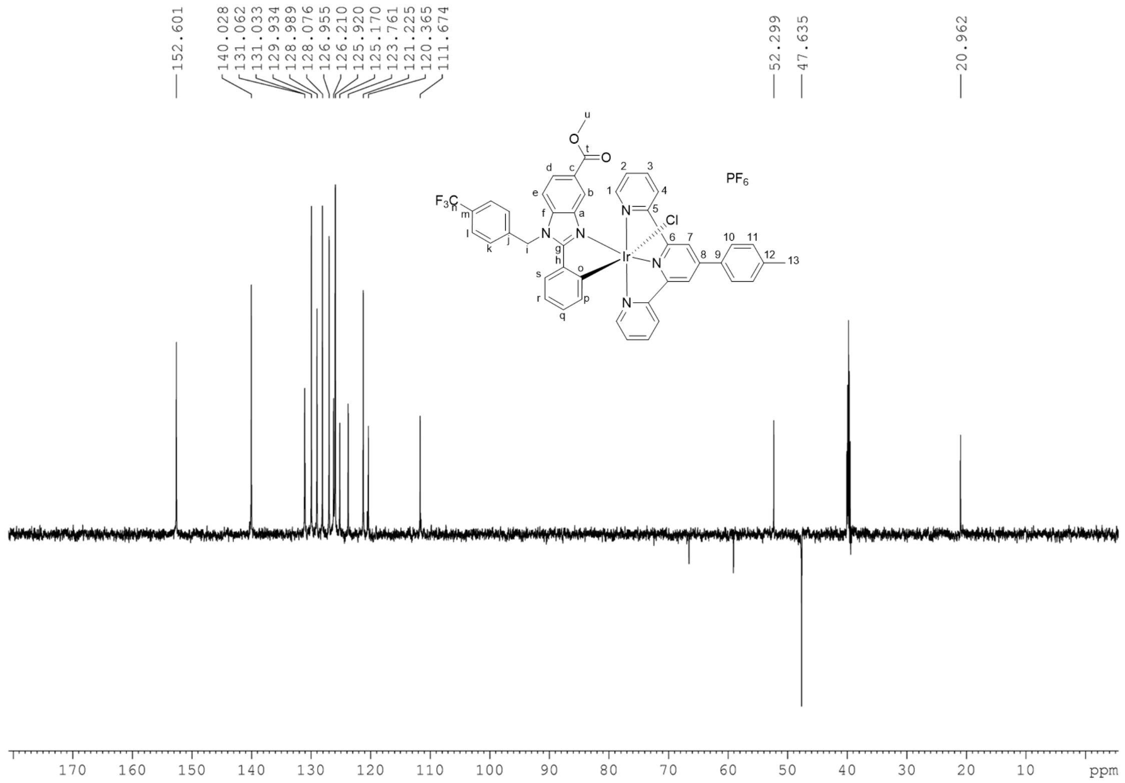

Figure S9. DEPT-135 NMR spectrum of **Ir10**, 151 MHz, DMSO-d_6.

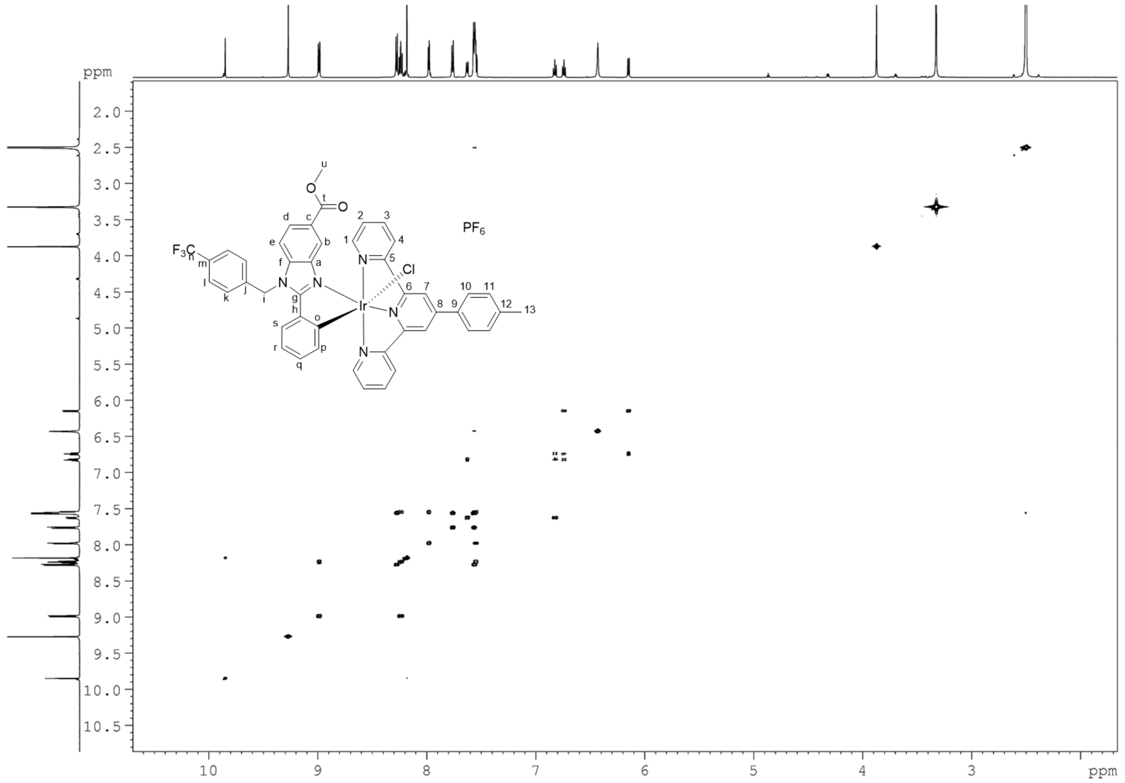

Figure S10. ^1H-^1H COSY NMR spectrum of **Ir10**, 600 MHz, DMSO-d_6.

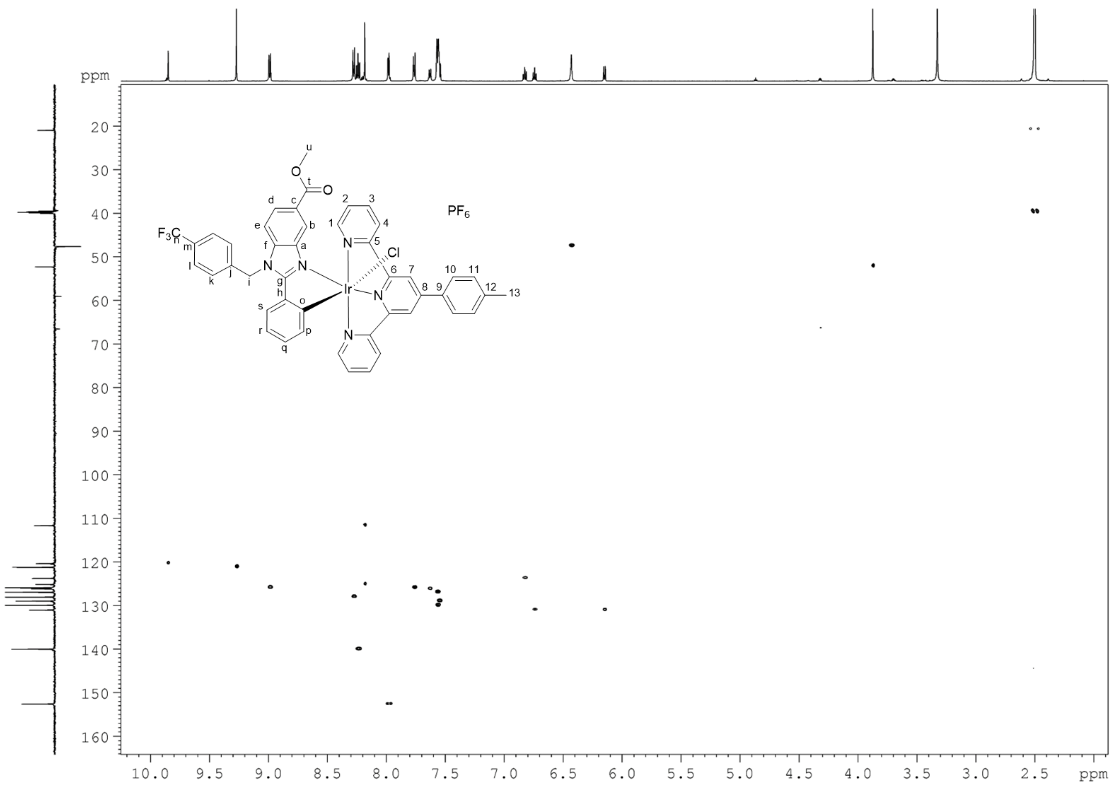

Figure S11. ^{1}H-^{13}C HSQC NMR spectrum of **Ir10**, 600 MHz, DMSO-d_6.

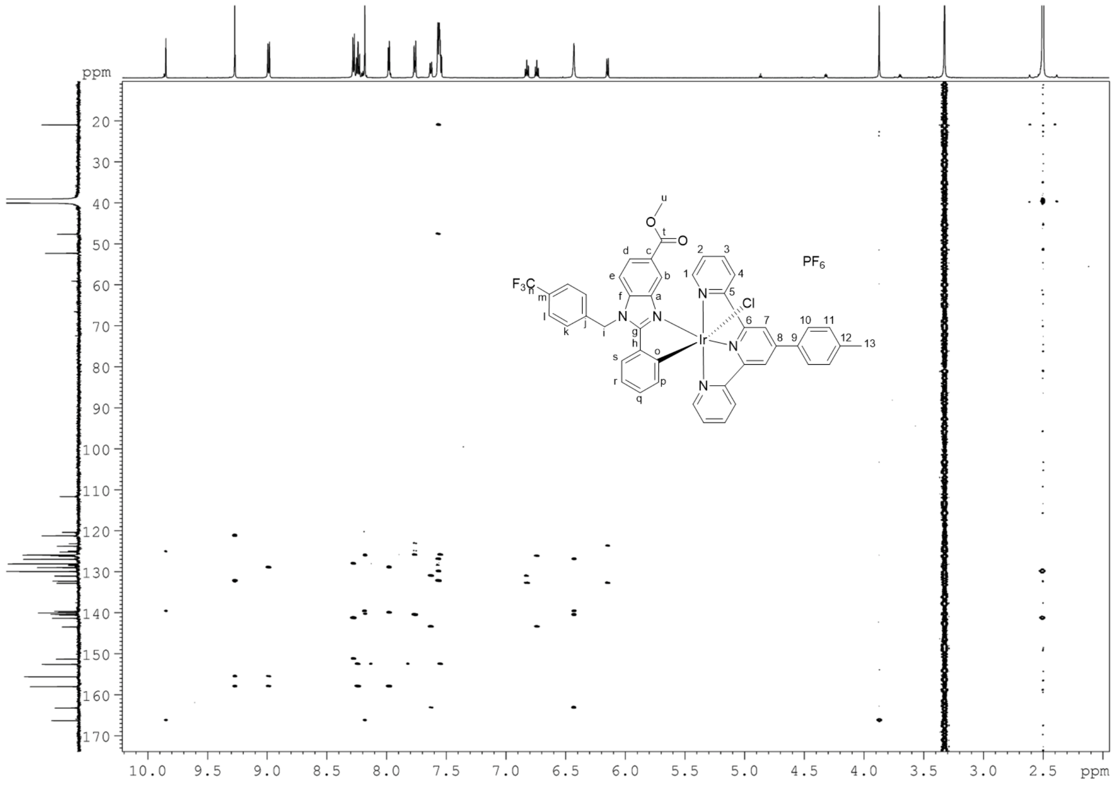

Figure S12. ^{1}H-^{13}C HMBC NMR spectrum of **Ir10**, 600 MHz, DMSO-d_6.

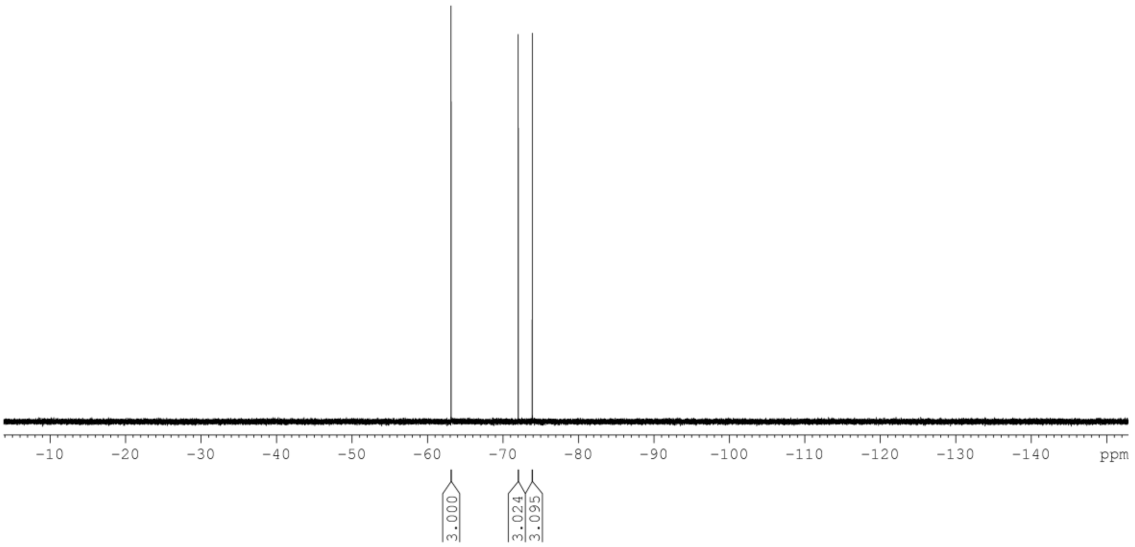

Figure S13. ^{19}F NMR spectrum of **Ir10**, 377 MHz, DMSO-d_6.

Mass spectrometry

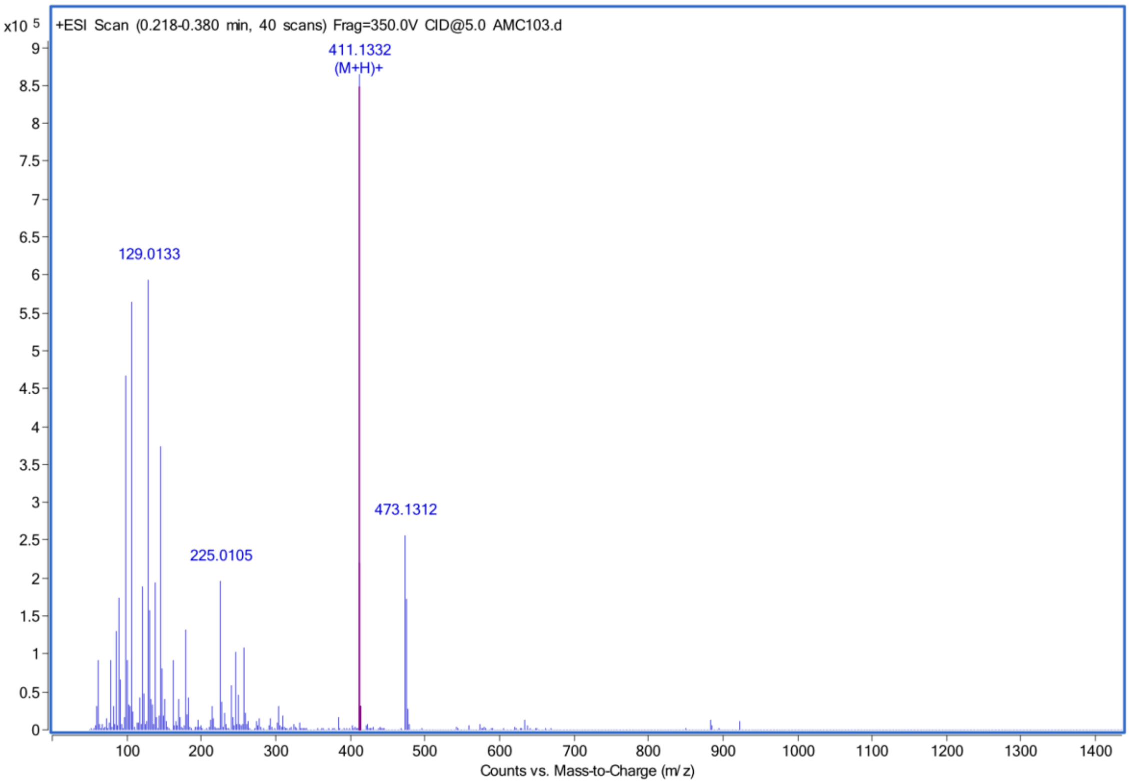

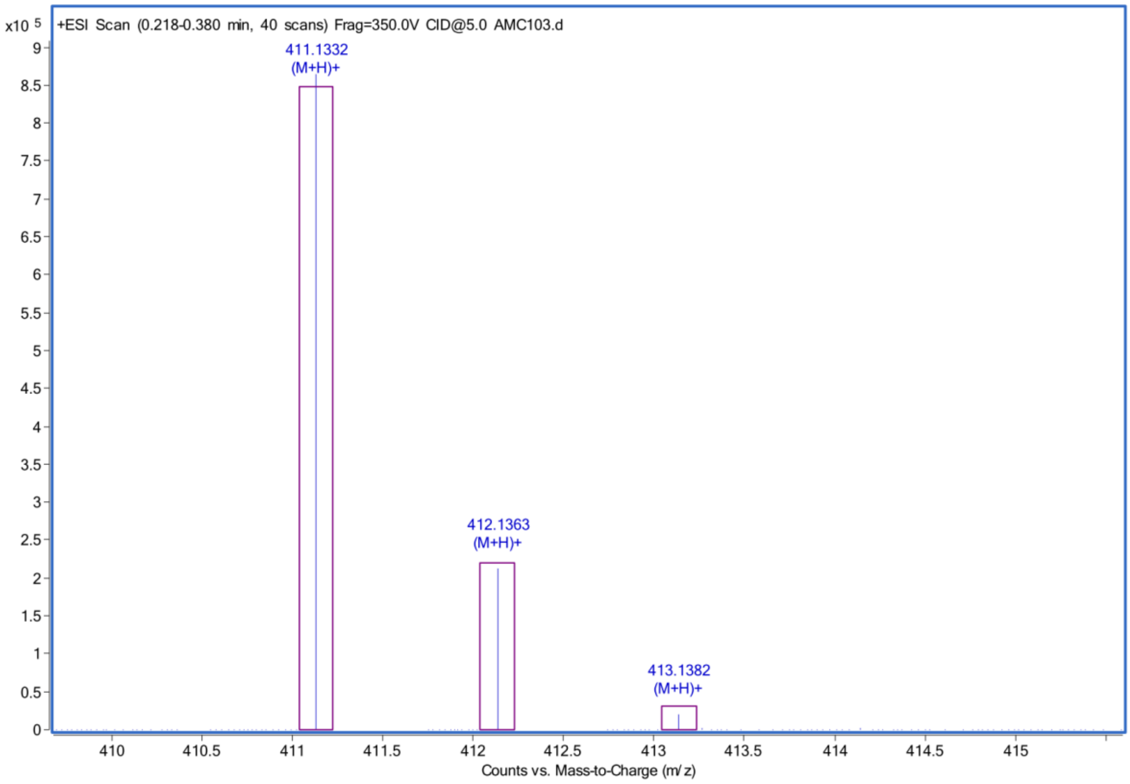

Figure S14. ESI-MS spectrum of **HL10** (positive detection mode).

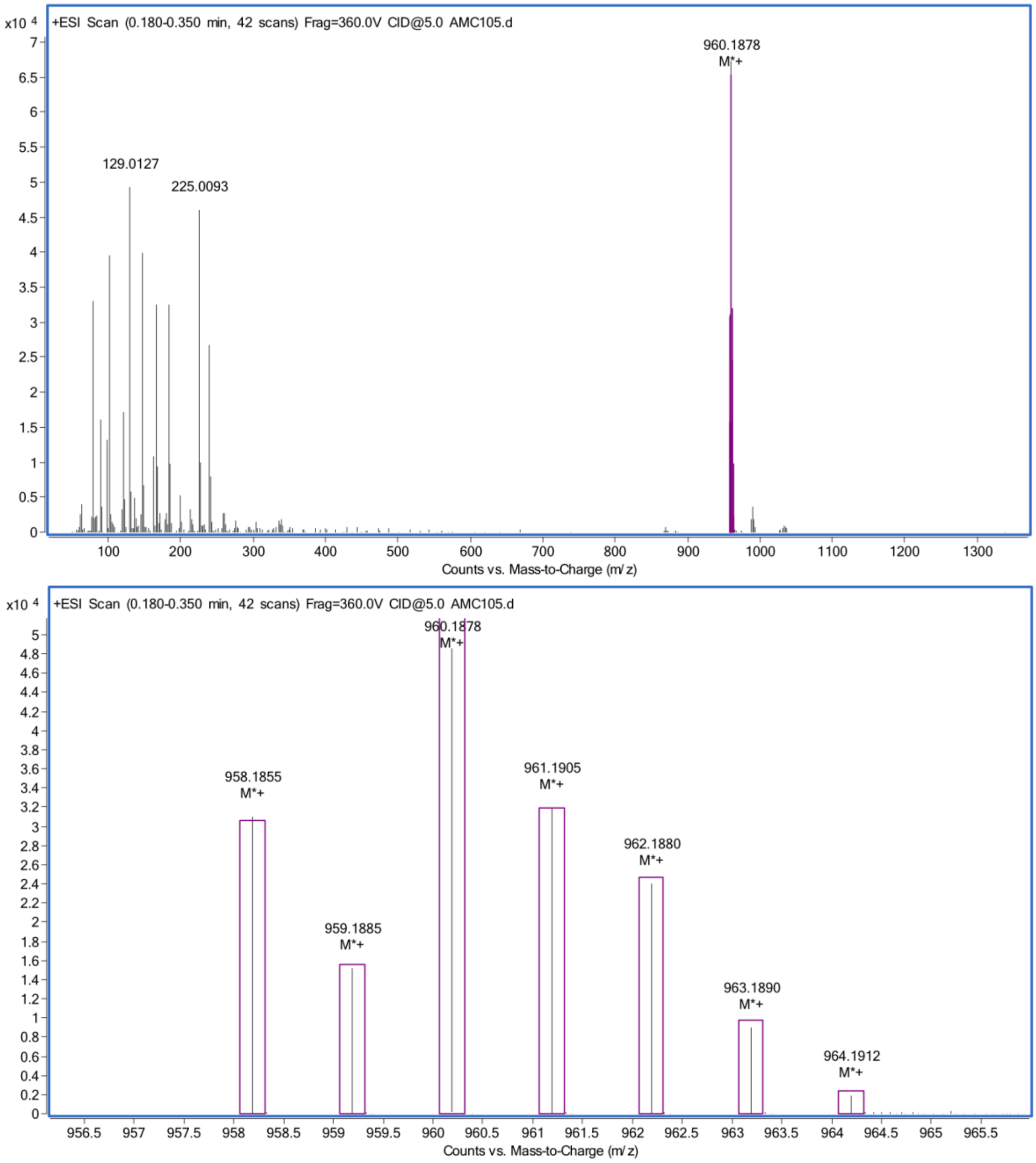

Figure S15. ESI-MS spectrum of **Ir10** (positive detection mode).

HPLC chromatograms.

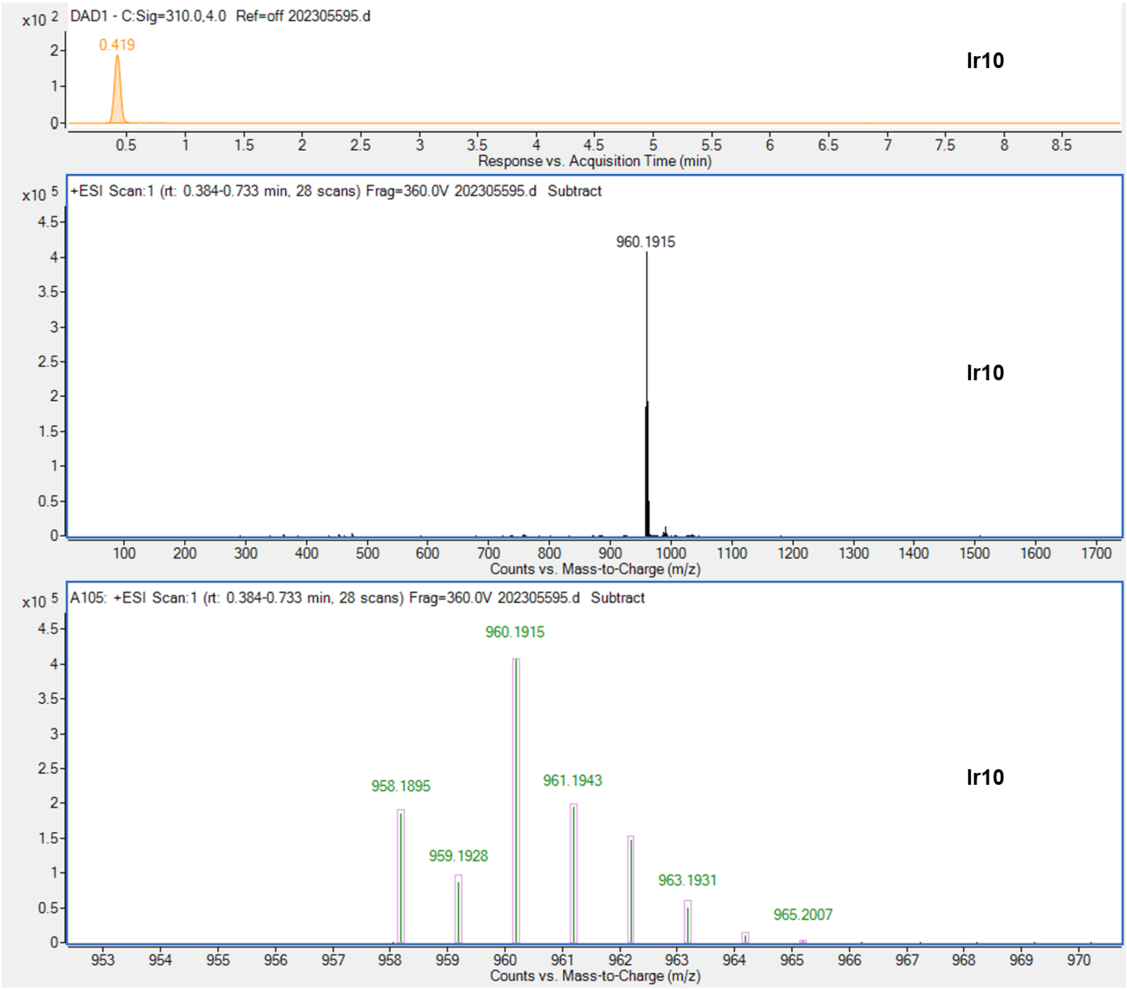

Figure S16. HPLC chromatograms with UV detection at 310 nm and the corresponding mass spectra of the 0.419 min peak for complex **Ir10**. Acetonitrile/methanol (0.1 % formic acid) (50/50) was used as the mobile phase.

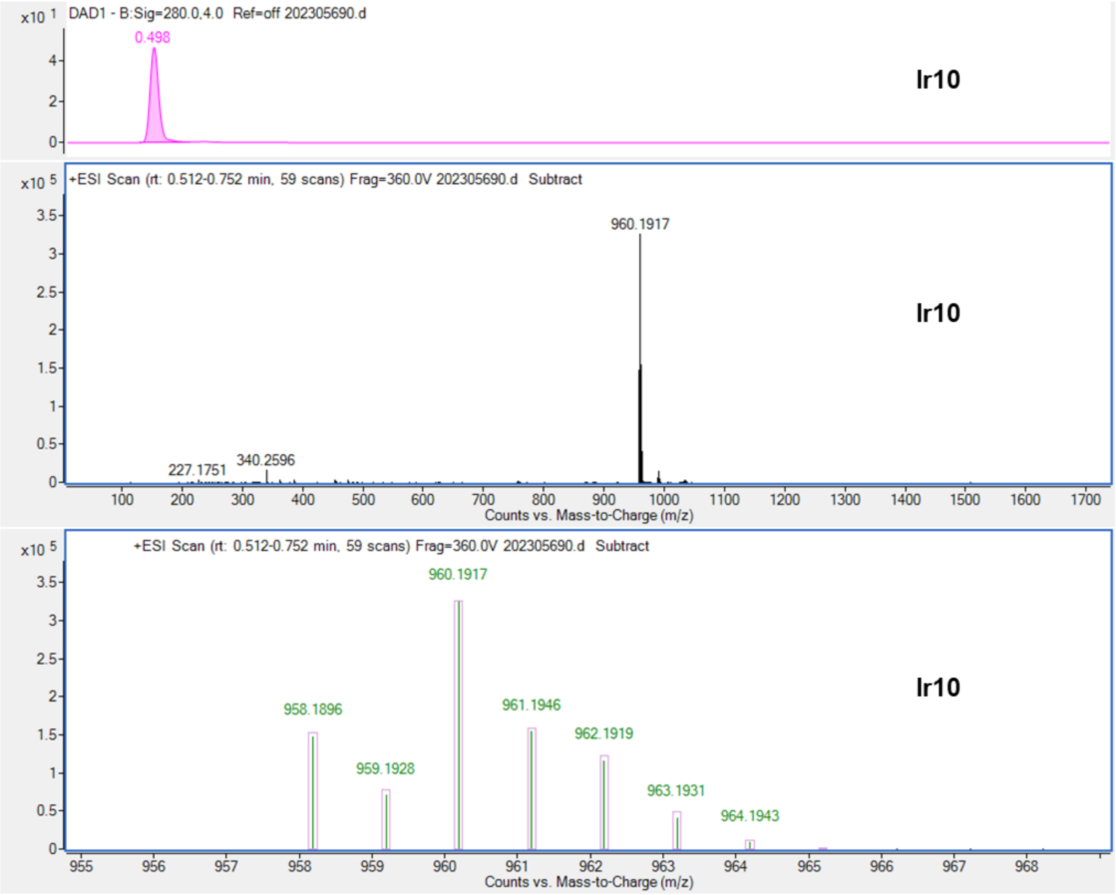

Figure S17. HPLC chromatogram of **Ir10** (UV detection at 280 nm) using acetonitrile:water (80:20) as a mobile phase in isocratic mode (0.1% formic acid) and the corresponding mass spectra showing the $[M\text{-}PF_6]^+$ peak.

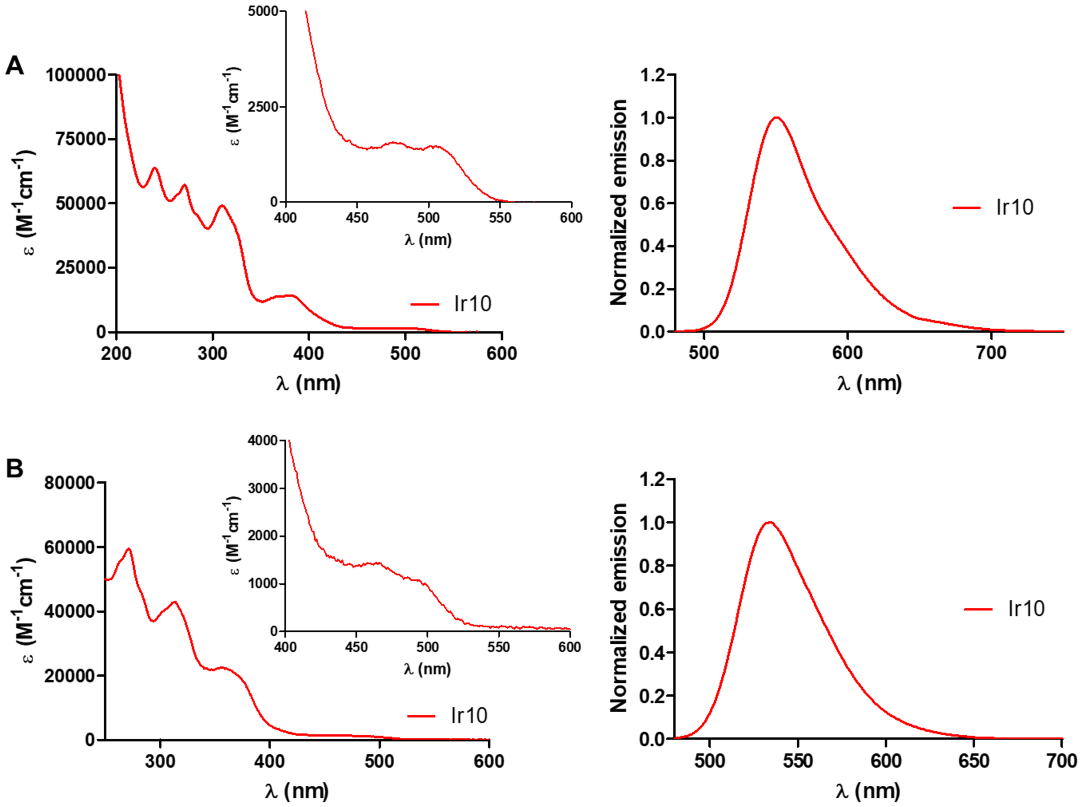

Figure S18. UV/Vis and emission (λ_{exc} = 405 nm) spectra of **Ir10** (10 µM) in aerated (A) acetonitrile and (B) water (1% DMSO).

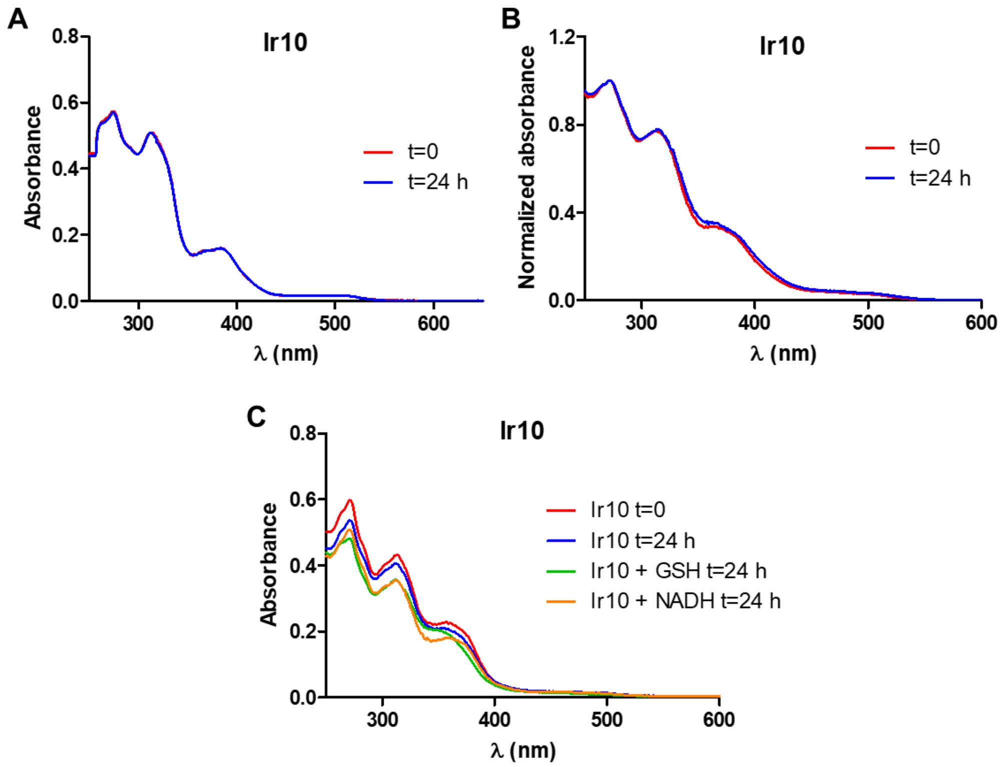

Figure S19. Time evolution of the absorbance spectrum of complex **Ir10** (10 µM) in (A) DMSO, (B) RPMI (5% DMSO), and (C) water (1% DMSO) in the absence and presence of GSH (10 mM) or NADH (100 µM).

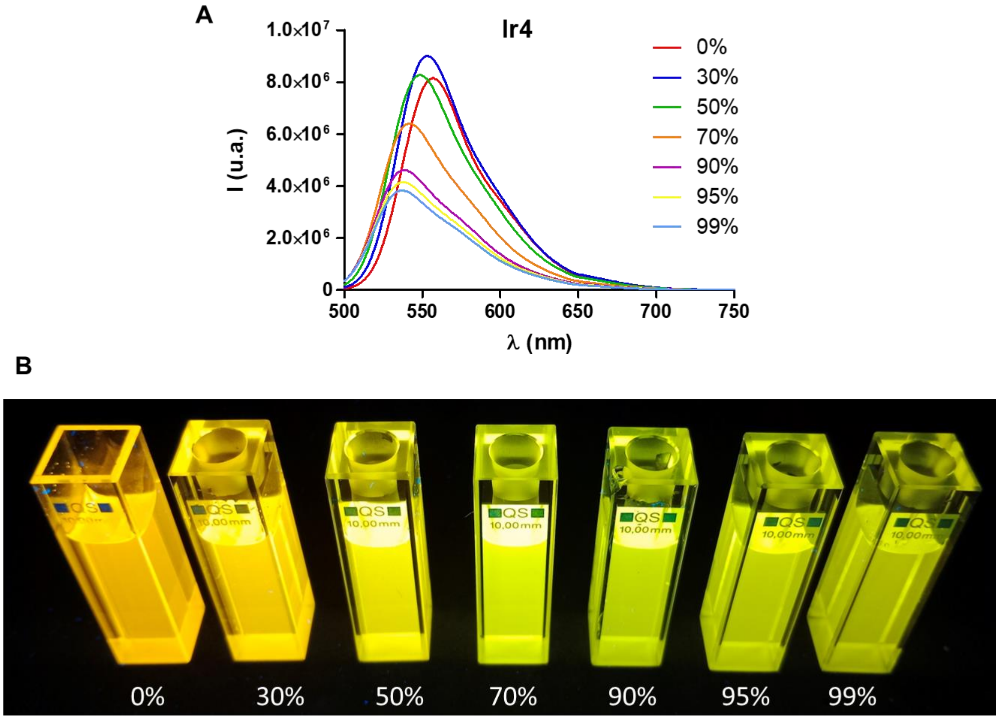

Figure S20. A) Emission spectra of complex **Ir4** (10 µM) in different water/DMSO mixtures. B) Photograph of the cuvettes containing the experiment under a 365 nm lamp.

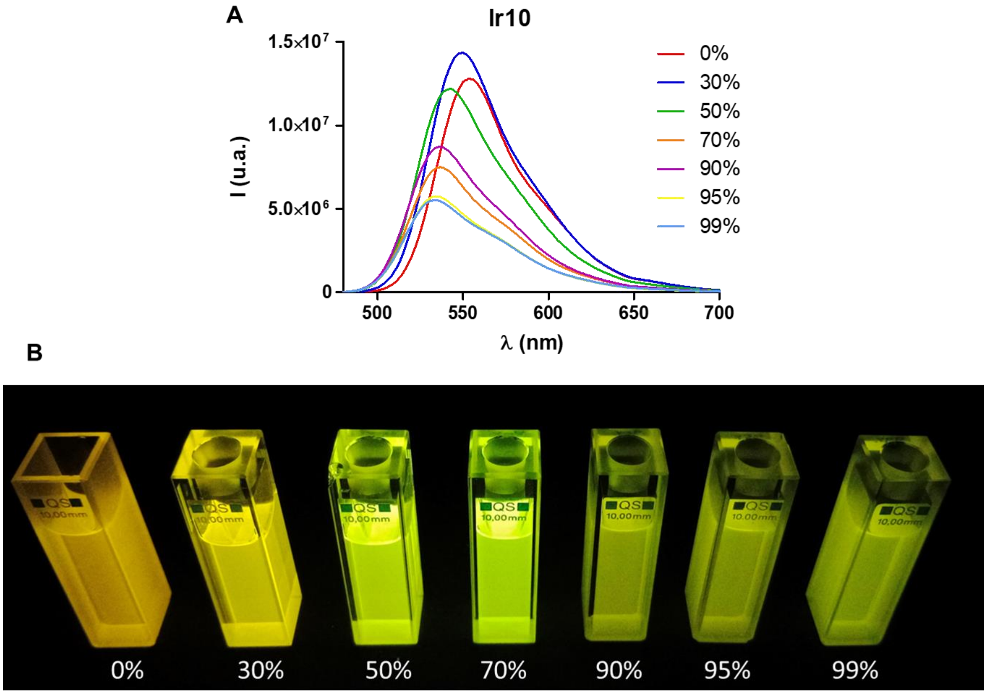

Figure S21. A) Emission spectra of complex **Ir10** (10 µM) in different water/DMSO mixtures. B) Photograph of the cuvettes containing the experiment under a 365 nm lamp.

Photostability

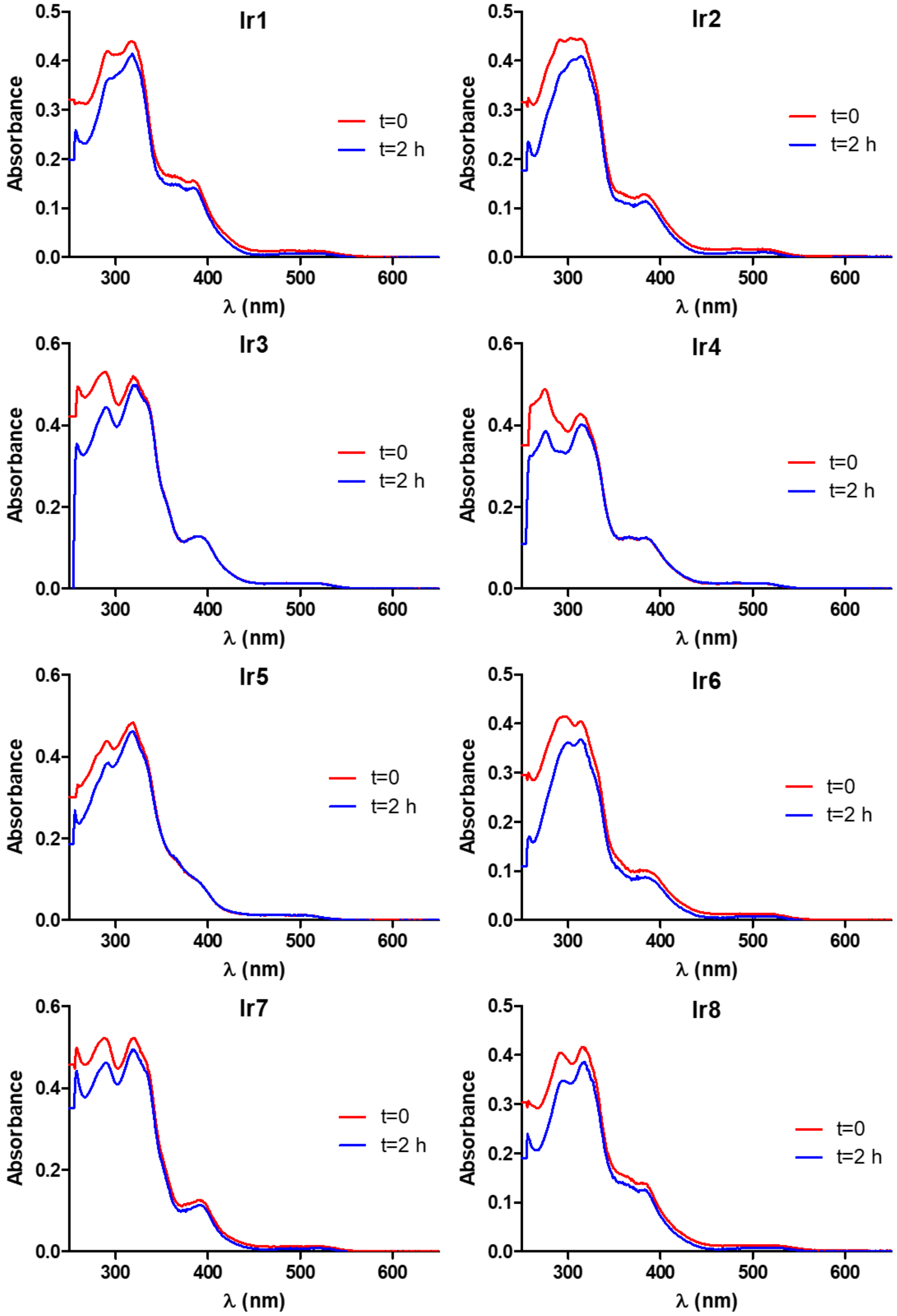

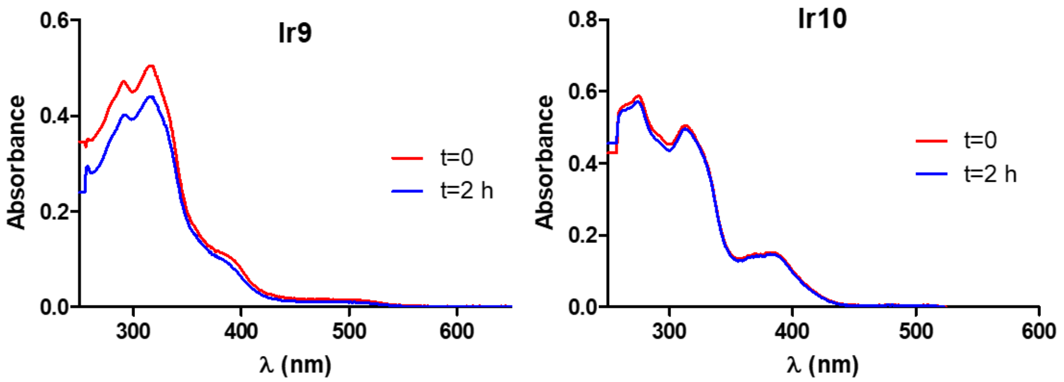

Figure S22. Photostability of the complexes **Ir1–Ir10** (10 µM) in DMSO by UV/Vis after 2 h of blue light irradiation (465 nm, 4.8 mW/cm^2).

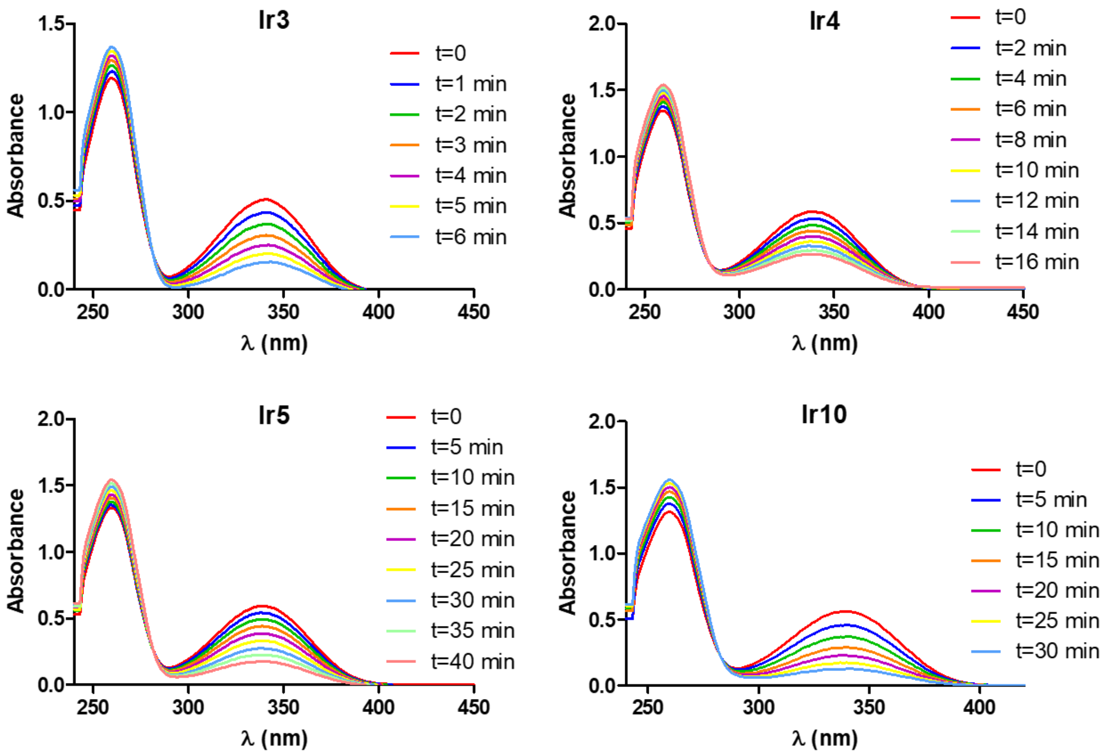

Figure S23. UV/Vis spectra for the photocatalytic oxidation of NADH (100 µM) by iridium complexes (5 µM) in PBS (5% DMSO) under blue light irradiation (λ = 465 nm, 4.8 mW/cm^2) at r.t.

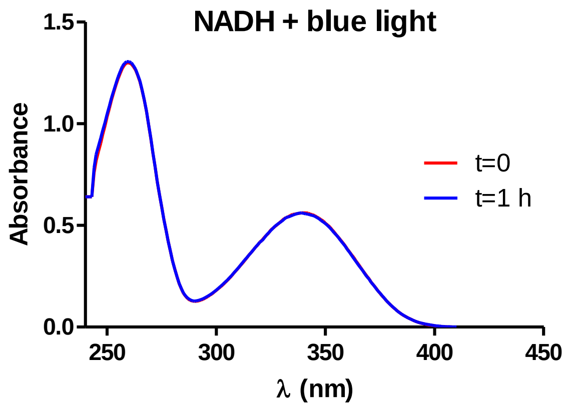

Figure S24. NADH UV/Vis spectra (100 µM) in PBS (5% DMF) under blue light irradiation (λ = 465 nm, 0.7 mW/cm^2) in the absence of complexes.

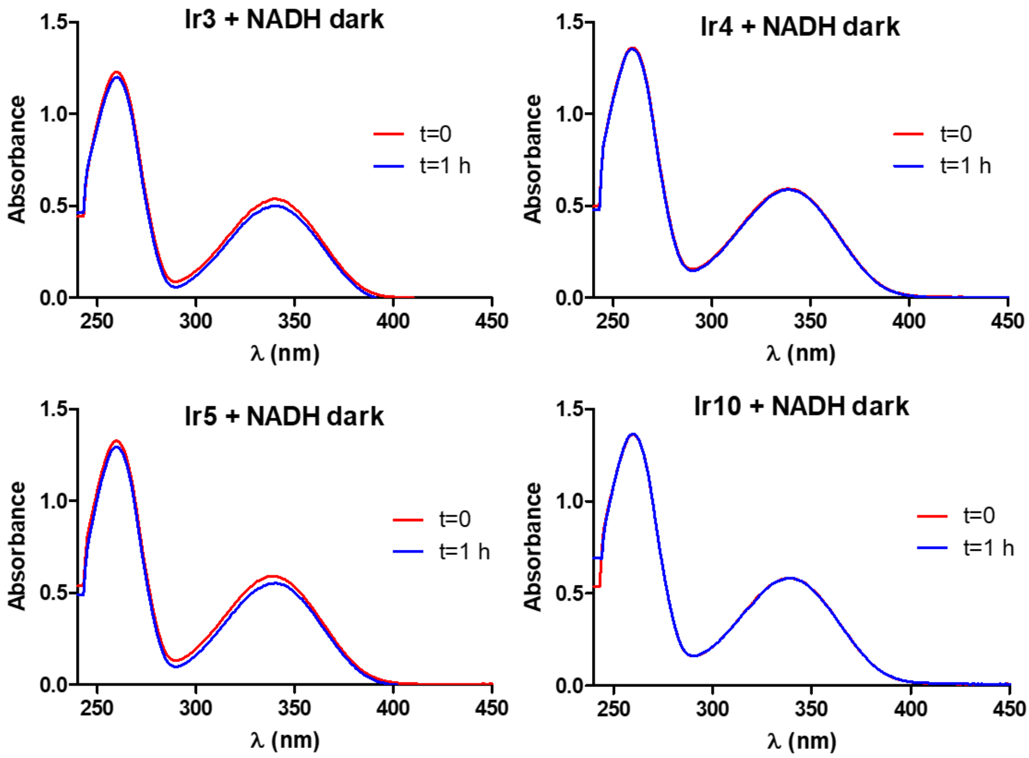

Figure S25. NADH UV/Vis spectra (100 µM) in the presence of the iridium complex (5 µM) in PBS (5% DMF) without light irradiation.

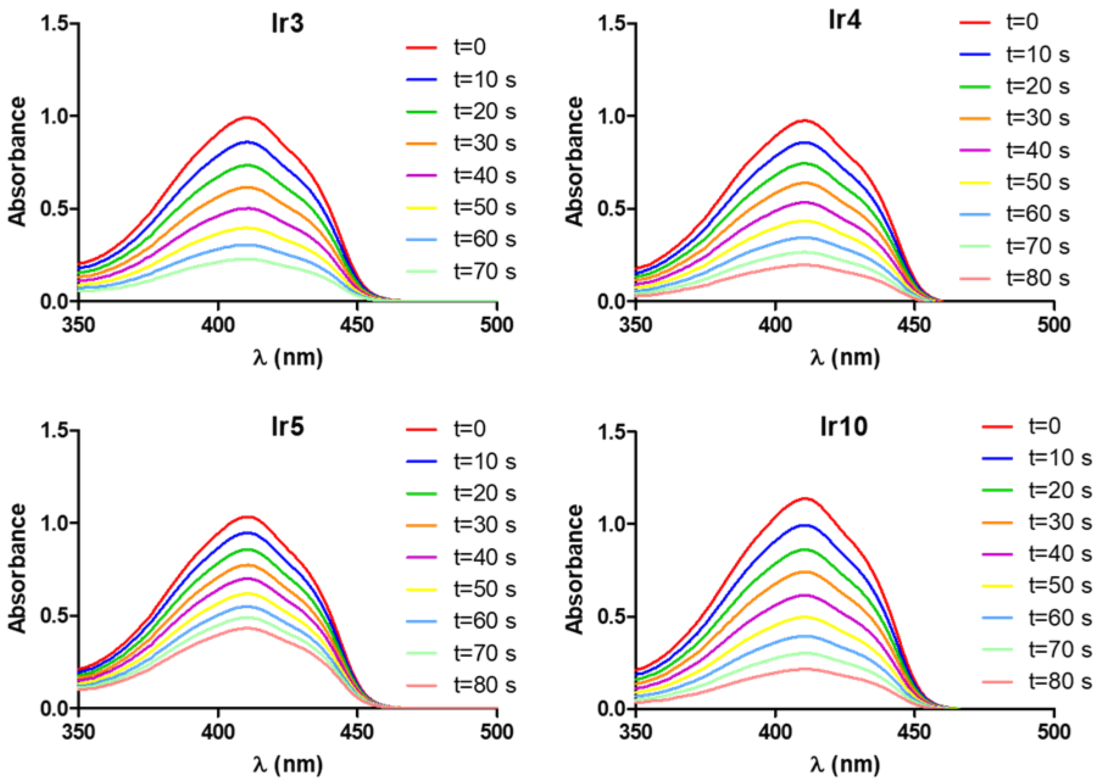

Figure S26. Evolution of the absorption spectra of DPBF in the presence of complexes **Ir3–Ir5** or **Ir10** in acetonitrile after blue light irradiation ($\lambda = 465$ nm, 0.7 mW/cm^2).

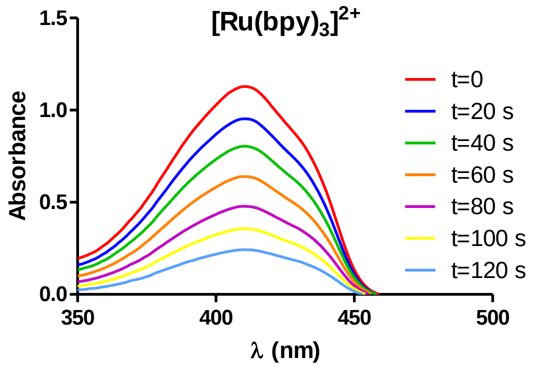

Figure S27. Evolution of the absorption spectrum of DPBF in the presence of the complex $[Ru(bpy)_3]^{2+}$ (used as reference) under blue light irradiation ($\lambda = 465$ nm, 0.7 mW/cm^2).

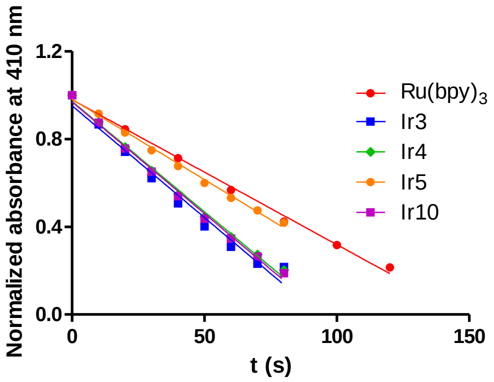

Figure S28. Absorbance of DPBF at 410 nm vs time (s) after the irradiation with blue light (λ = 465 nm, 0.7 mW/cm^2) in the presence of the different complexes in acetonitrile.

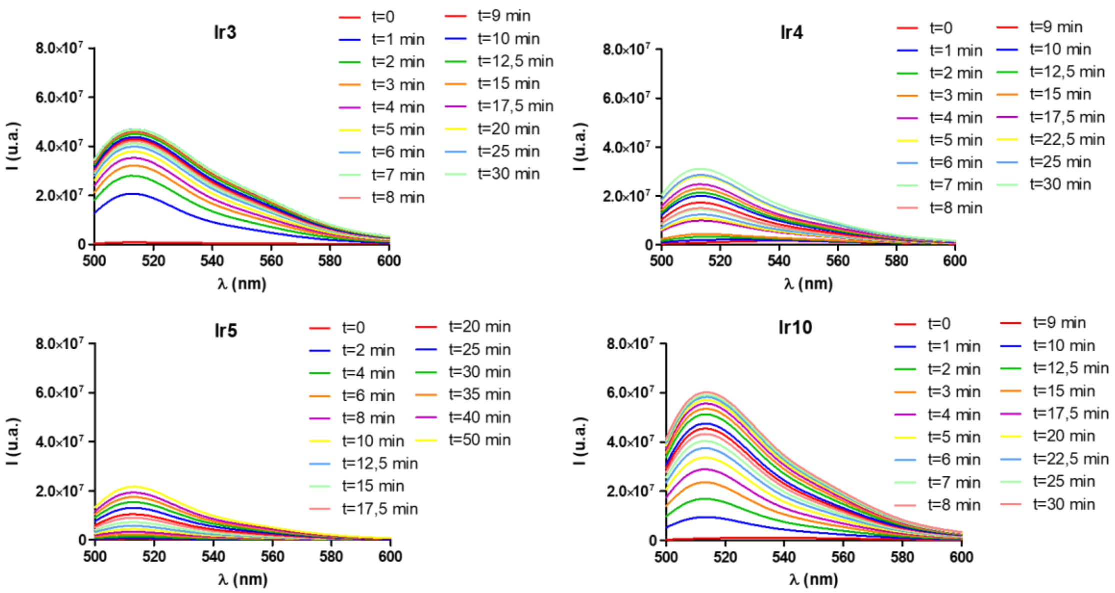

Figure S29. Evolution of the emission spectra of HPF (10 µM) in the presence of complexes **Ir3–Ir5 or Ir10** (10 µM) in PBS (5% DMF) under blue light irradiation (λ = 465 nm, 4.8 mW/cm^2), λ_{exc} = 490 nm.

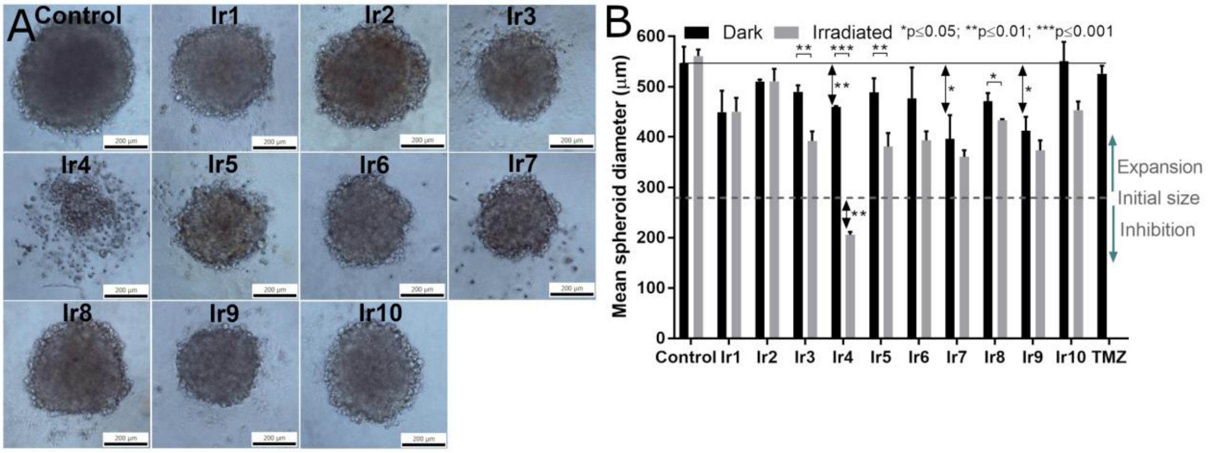

Figure S30. Analysis of the morphology of the brain glioblastoma U87MG spheroids treated with the investigated iridium complexes **Ir1** – **Ir10,** and temozolomide (TMZ). Spheroids were treated for 90 min with subsequent 30 min of irradiation with 420 nm blue light (58 Wm^{-2}) and then incubated for an additional 70 h in drug-free medium. Spheroids were formed under the ultra-low attachment 3D-forming conditions for 72 h. Phase contrast microphotographs were obtained for spheroids generated from U87MG cells (panel A), and the respective mean diameters were analyzed and shown in panel B for U87MG spheroids. The initial sizes of the spheroids at the starting point of the treatment are indicated using horizontal dashed lines in panel B. Data in panel B are the means from three independent experiments with quadruplicate of each data group, and the microphotographs in panel A show the representatives of spheroids treated with a concentration corresponding to IC_{50} for irradiated samples. Mean diameters for dark-incubated samples were analyzed at the same concentrations as those for irradiated samples to assess the impact of irradiation. Microphotographs in panel A represent the entire experimental set. All the data shown were obtained at the end of the 70 h drug-free incubation. Scale bars in panel A represent 200 µm. Statistical analysis was performed using Student's T-test, and the significance levels are marked as follows: $p \leq 0.05$*, 0.01**, 0.001***.

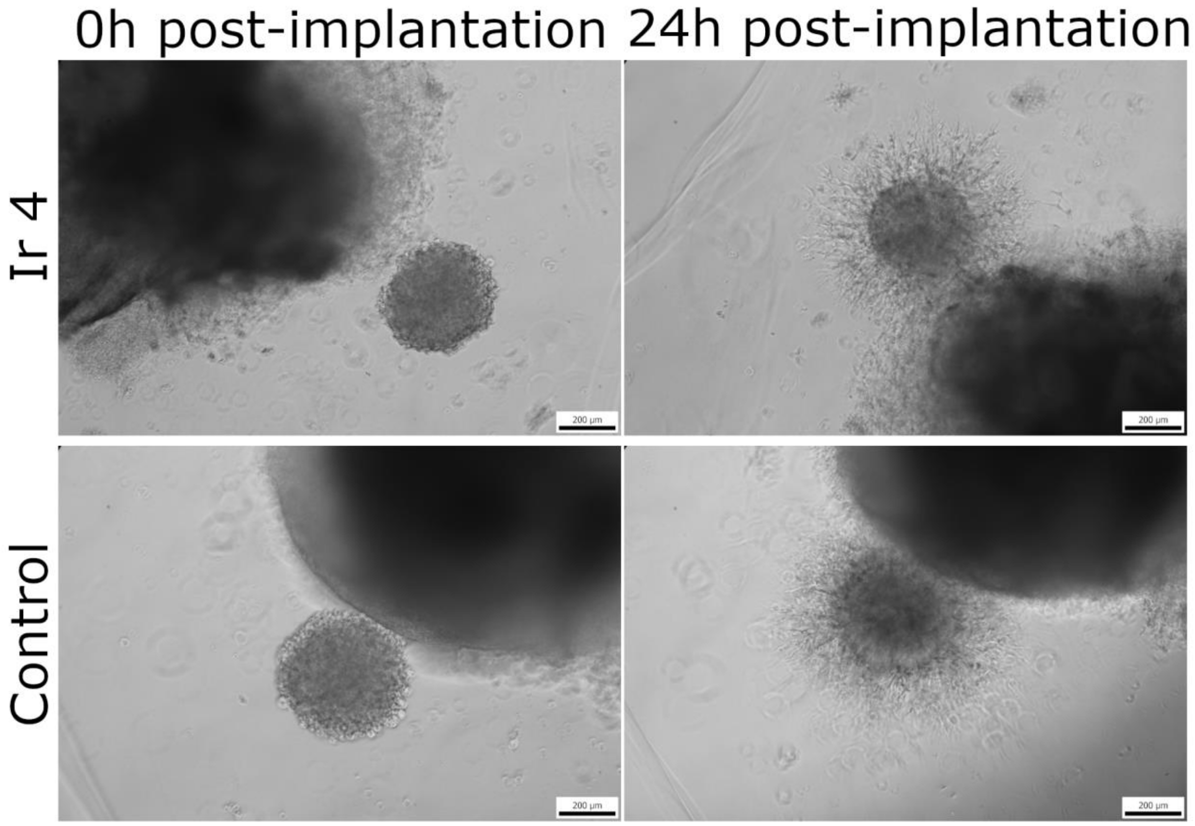

Figure S31. Example of organoid-spheroid implants immediately after implantation and 24 h post-implantation. A clearly visible invasion of U87MG glioblastoma cells could be observed. The scale bar represents 200 µm.

4. Schemes

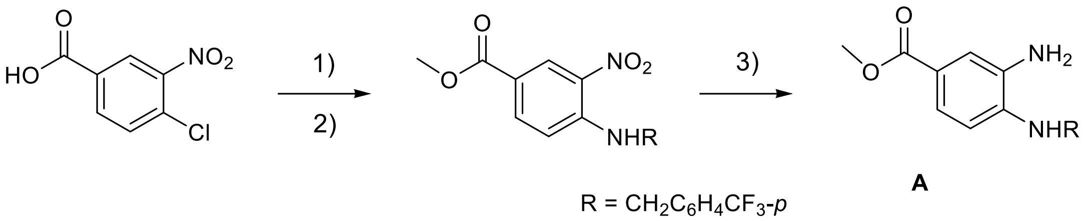

Scheme S1. Synthetic procedure for **A**. Reaction conditions: 1) H_2SO_4 (cat), MeOH, 65 °C, overnight; 2) *p*-$CF_3C_6H_4CH_2NH_2$, Et_3N, CH_2Cl_2, 72 h, N_2 atm., r.t.; 3) Zn (powder) $HCOONH_4$, AcOEt/MeOH N_2 atm., 24 h, 50 °C.

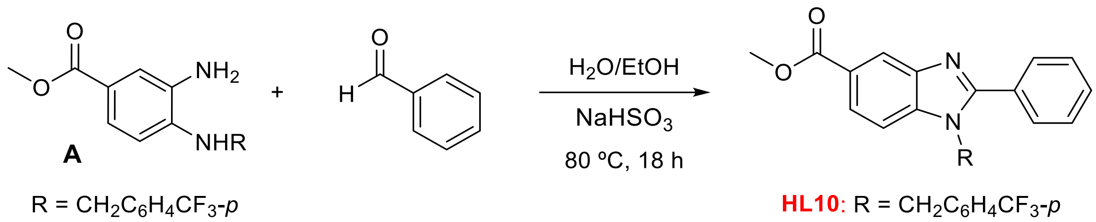

Scheme S2. Synthesis of proligand **HL10**.

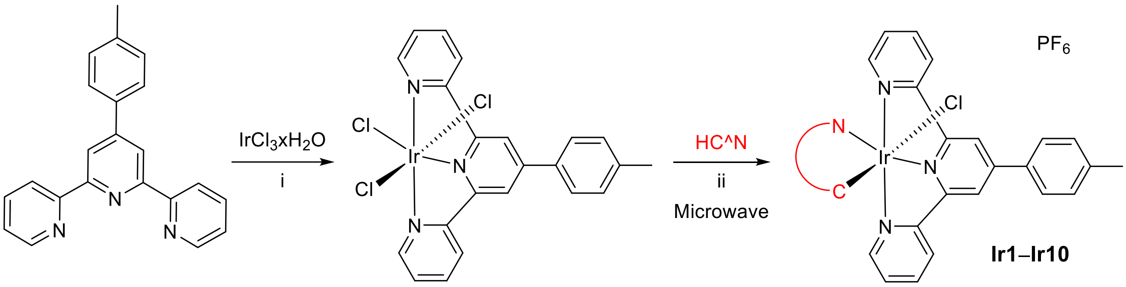

Scheme S3. Synthesis of iridium complex **Ir1-Ir10**: i) $IrCl_3xH_2O$ and ttpy in ethylene glycol at 180 °C for 25 min; ii) precursor complex and the HC^N proligand and KPF_6 in ethylene glycol at 240 °C for 12 min in a reaction microwave.

6. References

(1) Huang, H.; Banerjee, S.; Qiu, K.; Zhang, P.; Blacque, O.; Malcomson, T.; Paterson, M. J.; Clarkson, G. J.; Staniforth, M.; Stavros, V. G.; Gasser, G.; Chao, H.; Sadler, P. J. Targeted photoredox catalysis in cancer cells. *Nature Chem.* **2019**, *11*, 1041-1048.

(2) Kasparkova, J.; Hernández-García, A.; Kostrhunova, H.; Goicuría, M.; Novohradsky, V.; Bautista, D.; Markova, L.; Santana, M. D.; Brabec, V.; Ruiz, J. Novel 2-(5-arylthiophen-2-yl)-benzoazole cyclometalated iridium(III) dppz complexes exhibit selective phototoxicity in cancer cells by lysosomal damage and oncosis. *J. Med. Chem.* **2024**, *67*, 691-708.

(3) Zhuang, Z.; Dai, J.; Yu, M.; Li, J.; Shen, P.; Hu, R.; Lou, X.; Zhao, Z.; Tang, B. Z. Type I photosensitizers based on phosphindole oxide for photodynamic therapy: apoptosis and autophagy induced by endoplasmic reticulum stress. *Chem. Sci.* **2020**, *11*, 3405-3417.
